# Preventing recurrence in Sonic Hedgehog Subgroup Medulloblastoma using the OLIG2 inhibitor CT-179

**DOI:** 10.21203/rs.3.rs-2949436/v1

**Published:** 2023-06-09

**Authors:** Yuchen Li, Chaemin Lim, Taylor Dismuke, Daniel S. Malawsky, Sho Oasa, Zara C. Bruce, Carolin Offenhäuser, Ulrich Baumgartner, Rochelle C. J. D’Souza, Stacey L. Edwards, Juliet D. French, Lucy S.H. Ock, Sneha Nair, Haran Sivakumaran, Lachlan Harris, Andrey P. Tikunov, Duhyeong Hwang, Coral Del Mar Alicea Pauneto, Mellissa Maybury, Timothy Hassall, Brandon Wainwright, Santosh Kesari, Gregory Stein, Michael Piper, Terrance G. Johns, Marina Sokolsky-Papkov, Lars Terenius, Vladana Vukojević, Timothy R. Gershon, Bryan W. Day

**Affiliations:** 1 QIMR Berghofer Medical Research Institute, Brisbane, QLD, 4006, Australia.; 2 These authors contributed equally.; 3 The University of Queensland, Brisbane, QLD, 4072, Australia.; 4 Center for Nanotechnology in Drug Delivery and Division of Pharmacoengineering and Molecular Pharmaceutics, Eshelman School of Pharmacy, University of North Carolina at Chapel Hill, NC 27599, USA.; 5 College of Pharmacy, Chung-Ang University, 221 Heukseok-dong, Dongiak-gu, Seoul 06974, Republic of Korea.; 6 Department of Neurology, University of North Carolina School of Medicine, Chapel Hill, NC, 27599, USA.; 7 Wellcome Sanger Institute, Hinxton, Cambridgeshire, UK.; 8 Department of Clinical Neuroscience, Center for Molecular Medicine (CMM), Karolinska Institutet, 17176 Stockholm, Sweden; 9 School of Biomedical Sciences, Queensland University of Technology, Brisbane, QLD, 4072, Australia.; 10 Department of Pediatrics, Emory University, Atlanta, GA 30323, USA; 11 Department of Pharmaceutical Engineering, Dankook University, 119 Dandae-ro, Dongnam-gu, Cheonan 31116, Republic of Korea.; 12 Department of Pharmacology, University of North Carolina School of Medicine, Chapel Hill, NC, 27599, USA; 13 Child Health Research Centre, The University of Queensland, Brisbane, QLD, 4101, Australia.; 14 Oncology Service, Queensland Children’s Hospital, Children’s Health Queensland Hospital & Health Service, Brisbane, QLD, 4101, Australia.; 15 Curtana Pharmaceuticals, Inc. Austin, TX 78756, United States.; 16 Telethon Kids Institute, Perth, WA, 6009, Australia.; 17 Lead contact

**Keywords:** Medulloblastoma, OLIG2, CT-179, pre-clinical

## Abstract

Recurrence is the primary life-threatening complication for medulloblastoma (MB). In Sonic Hedgehog (SHH)-subgroup MB, OLIG2-expressing tumor stem cells drive recurrence. We investigated the anti-tumor potential of the small-molecule OLIG2 inhibitor CT-179, using SHH-MB patient-derived organoids, patient-derived xenograft (PDX) tumors and mice genetically-engineered to develop SHH-MB. CT-179 disrupted OLIG2 dimerization, DNA binding and phosphorylation and altered tumor cell cycle kinetics *in vitro* and *in vivo*, increasing differentiation and apoptosis. CT-179 increased survival time in GEMM and PDX models of SHH-MB, and potentiated radiotherapy in both organoid and mouse models, delaying post-radiation recurrence. Single cell transcriptomic studies (scRNA-seq) confirmed that CT-179 increased differentiation and showed that tumors up-regulated *Cdk4* post-treatment. Consistent with increased CDK4 mediating CT-179 resistance, CT-179 combined with CDK4/6 inhibitor palbociclib delayed recurrence compared to either single-agent. These data show that targeting treatment-resistant MB stem cell populations by adding the OLIG2 inhibitor CT-179 to initial MB treatment can reduce recurrence.

## INTRODUCTION

Brain cancer remains the leading cause of cancer-related death in children [1]. Medulloblastoma (MB) is the most common pediatric brain cancer and accounts for approximately 20% of all cases [2]. International consensus recognises four distinct MB molecular subgroups: Wingless (WNT), Sonic hedgehog (SHH), Group 3 and Group 4 tumors [3]. Overall survival (OS) rates have reached 70–80%, but the outcome for young children, especially infants, is worse [4, 5]. Those who do survive suffer from long-term therapy-induced side effects and development of secondary tumors [6–10]. Novel therapies with specific anti-tumor efficacy and less off-target toxicity hold promise to improve both survival rates and quality of life for MB patients.

OLIG2 is a basic helix-loop-helix (bHLH) transcription factor that functions in the developing brain in a bivalent manner, promoting differentiation in the oligodendrocyte lineage while maintaining neural stem and progenitor cells in an undifferentiated state [11–13]. OLIG2 phosphorylation plays a critical role in determining this bivalent function [14, 15]. In brain tumors, the anti-differentiation function of OLIG2 plays an important role in tumor progression, as seen in glioblastoma (GBM) [16] and in SHH MB [17]. In SHH MB, moreover, OLIG2-expressing stem cells promote recurrence after cytotoxic chemotherapy and radio-resistance [18]. These data support a model in which MB stem cells within this heterogeneous tumor microenvironment are inadequately treated by standard therapy and drive recurrence.

Considering the role of OLIG2 in restricting neural stem cell differentiation, and the role of OLIG2-expressing tumor stem cells in SHH-MB recurrence, we investigated whether pharmacologic disruption of OLIG2 would enhance MB therapy by targeting stem cells that may be resistant to radiation and cytotoxic chemotherapy. As OLIG2+ cells make up only a fraction of the proliferative population within MB, we expected that OLIG2 inhibitor therapy would require combination with additional therapies for optimal anti-tumor effect. We therefore investigated the efficacy of anti-OLIG2 therapy in both single agent format, to define potential anti-tumor effects, and in multi-modal combinations as are typically needed in clinical treatment of MB patients.

OLIG2, like other bHLH transcription factors, requires dimerization to initiate function [19]. Therefore, this dimerization interface presents an opportunity for targeted disruption [20]. A series of small molecule OLIG2 inhibitors were previously identified using a pharmacophore-guided 3D-structural search to find compounds that engage the OLIG2 dimerization interface [21]. The ability of these compounds to disrupt OLIG2 dimerization was demonstrated and quantitatively measured using fluorescence cross-correlation spectroscopy (FCCS), which measures the temporal correlation between signals from OLIG2 molecules individually tagged with either eGFP or Tomato, after co-transfection [22]. These methods showed that the disruptive effects of different agents on OLIG2 dimerization correlated with the inhibitory effects of these agents on *in vitro* tumor cell growth [21, 22], supporting the proposed mechanism of action of these agents and the specificity of their anti-tumor effect.

In this study we analyzed OLIG2 expression in MB subgroups and evaluated the effect of OLIG2 inhibition on tumor growth, either as a single intervention and in combinatorial therapies. We investigated OLIG2 inhibition using CT-179, a compound based on the previously studied agent SKOG102. CT-179 is a novel small molecule OLIG2 inhibitor developed by Curtana Pharmaceuticals (molecular weight (MW) 397.3 g/mol, patent application WO2016138479A1) that has shown favorable blood brain barrier (BBB) penetration and stability in preliminary animal studies. CT-179 was recently awarded FDA Rare Pediatric Disease Designation for the treatment of MB.

We evaluated CT-179 using MB cell lines, explant tissue organoids (MBOs) and *in vivo* models that are prone to recurrence, including patient-derived xenografts (PDX) and a genetically-engineered mouse (GEM) model, analyzed using FCCS, kinomic analysis, *in vitro* and *in vivo* cell cycle analysis, *in vivo* tumor treatment studies and scRNA-seq. Our findings validate the potential of targeting OLIG2 in SHH-MB and highlight the novel brain penetrant small molecule CT-179 for further clinical evaluation, particularly when combined with radiotherapy (RT) in patients with MB.

## RESULTS

### OLIG2 correlates with poor survival in SHH-MB patients

**Figure 1.**
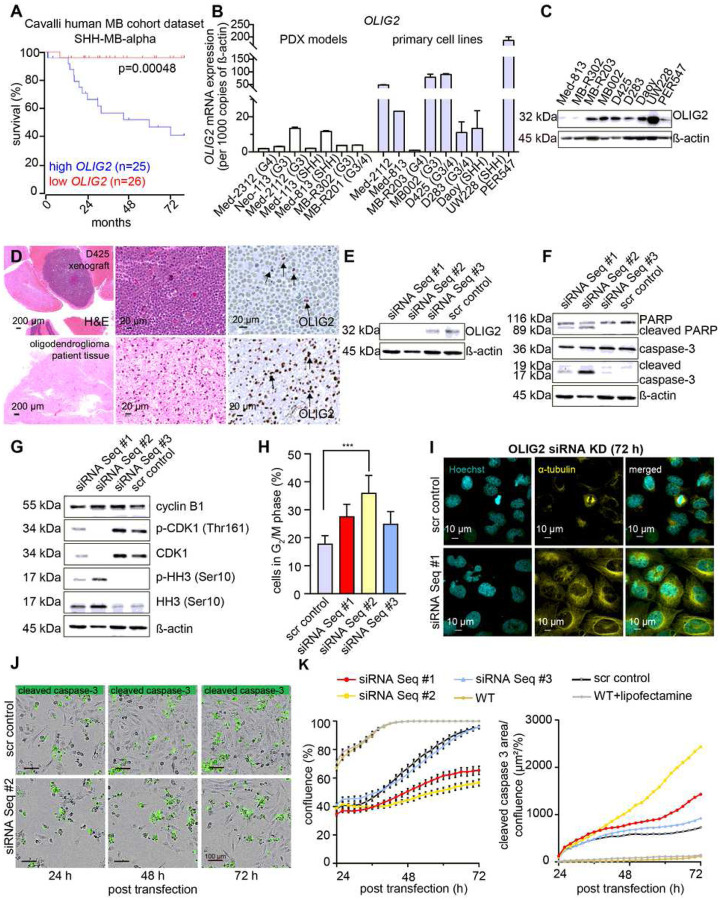
OLIG2 expression in pediatric MB and OLIG2 siRNA knockdown studies (A) High *OLIG2* expression correlates with poor OS in SHH-MB α subtype patients which harboring *TP53* mutation. Kaplan-Meier curves of the SHH-driven MB α subtype patients based on OLIG2 expression. (B) *OLIG2* expression in PDX models, primary cell lines and ATCC pediatric MB cell lines (data are shown as means ± SD, n=6). (C) OLIG2 expression at protein level in MB cell lines. (D) OLIG2 immunostaining in a MB xenograft model D425. Oligodendroglioma patient tissue was used as a positive control. Arrowheads, OLIG2^+^ cells. Scale bars are indicated in (D) (E) Representative western blots show expression of OLIG2 expression 72 hours after siRNA OLIG2 knockdown (KD) in Daoy cells. (F) Representative western blots show expression of cleaved caspase-3, total caspase-3 and cleaved PARP and total PARP in Daoy 72 hours after siRNA OLIG2 knockdown. (G) Representative western blots show expression of cyclin B1, p-CDK1, p-HH3, total CDK1 and total HH3 in Daoy 72 hours after siRNA OLIG2 knockdown. (H) Daoy cells stalled in G_2_/M phase 72 hours after siRNA OLIG2 knockdown (means ± SD, **p < 0.01, n=6). The p value was determined by one-way ANOVA. (I) Top panel shows a Daoy cell in anaphase with normal nucleus morphology and spindle alignment. siRNA OLIG2 knockdown results in abnormal nucleus phenotypes including satellite micronuclei. Scale bars are indicated in (I). (J) Bright field images of Daoy cells transfected with scrambled control sequence and siRNA OLIG2 KD Seq #2 over 48 hours. Cells labelled with cleaved Caspase-3/7 were shown in green. (K) Cell confluence (left) and cleaved caspase-3 confluence of Daoy after siRNA knockdown compared to scrambled control, wild-type Daoy with or without lipofectamine.

Analysis of OLIG2 expression supported a significant role in SHH-MB heterogeneity. Previous studies have shown that *OLIG2* significantly correlated with patient outcome in SHH-MB patients [17]. To investigate the relationship of *OLIG2* to survival in SHH-MB in specific subtypes, we analyzed transcriptomic datasets previously published by Cavalli and colleagues [23]. Results show that *OLIG2* expression correlated with for poor patient outcomes in alpha, delta and gamma subtypes of SHH-MB, reaching significance for the alpha and gamma patient cohorts (Figure 1A, Supplementary Figure 1A). Next, we analysed *OLIG2* mRNA expression in a panel of immortalized MB lines, primary MB lines and PDX models [24]. *OLIG2+* cell lines showed more abundant *OLIG2* mRNA compared to *OLIG2+* PDX tumors, consistent with heterogeneous expression in the tumors (Figure 1B, Supplementary Figure 1B). Western blots confirmed translation of *OLIG2* mRNA in MB PDX and cell lines (Figure 1C). In addition, immunohistochemistry (IHC) staining for OLIG2, performed on MB xenograft tumors, showed positive focal OLIG2 staining (Figure 1D, Supplementary Figure 1C). Taken together, these findings highlight a potential role for OLIG2 in both inter- and intra-tumoral heterogeneity.

### OLIG2 down-regulation induces G_2_/M phase cell cycle arrest and apoptosis

For initial analysis of OLIG2 function in MB, we silenced *OLIG2* using small interfering RNAs (siRNA) in the SHH MB-derived Daoy cell line and analyzed the effects on growth kinetics and cell death *in vitro*. 72 hours post transfection, robust OLIG2 expression in untreated cells and effective knockdown (KD) in siRNA-treated cells were shown at the mRNA (Supplementary Figure 1D) and protein level (Figure 1E). Low-level OLIG2 protein expression persisted in cells treated with sequence #3 but OLIG2 was undetectable in cells treated with sequence #1 and #2. The degree of OLIG2 silencing correlated with the observed apoptotic response with cell death observed with sequence #1 and #2 but not sequence #3 (Figure 1F).

Prior studies show OLIG2-dependent regulation of multiple cell cycle-associated transcriptional regulators and genes controlling microtubule function during development [25, 26]. We therefore assessed the cell cycle following OLIG2 KD. OLIG2 KD disrupted cell cycle progression, resulting in decreased cyclin dependent kinase 1 (CDK1) and phosphorylated CDK1 (p-CDK1) and increased expression of M-phase specific marker phosphorylated histone H3 (p-HH3) (Figure 1G). Consistent with these changes, down-regulation of OLIG2 led to an increased proportion of cells in G_2_/M phase (Figure 1H). OLIG2 KD also altered nuclear morphology, demonstrated by Hoescht (cyan) and altered alignment, demonstrated by α-tubulin immunofluorescence (IF) (yellow). In contrast, after OLIG2 KD, cells showed a multinucleated/tetraploid appearance (Figure 1I). Cell proliferation and apoptosis were assessed following KD using real-time live cell imaging; representative images are shown in Figure 1J and Supplementary Figure 1E. Quantification showed significant anti-proliferative and apoptotic effects using sequence #1 and #2 but less so using the less effective sequence #3 (Figure 1K). These data demonstrate an important role of OLIG2 in the regulation of MB cell cycle progression and apoptosis, suggesting the therapeutic potential of targeting OLIG2.

### CT-179 disrupts OLIG2 function by interfering with dimerization.

**Figure 2.**
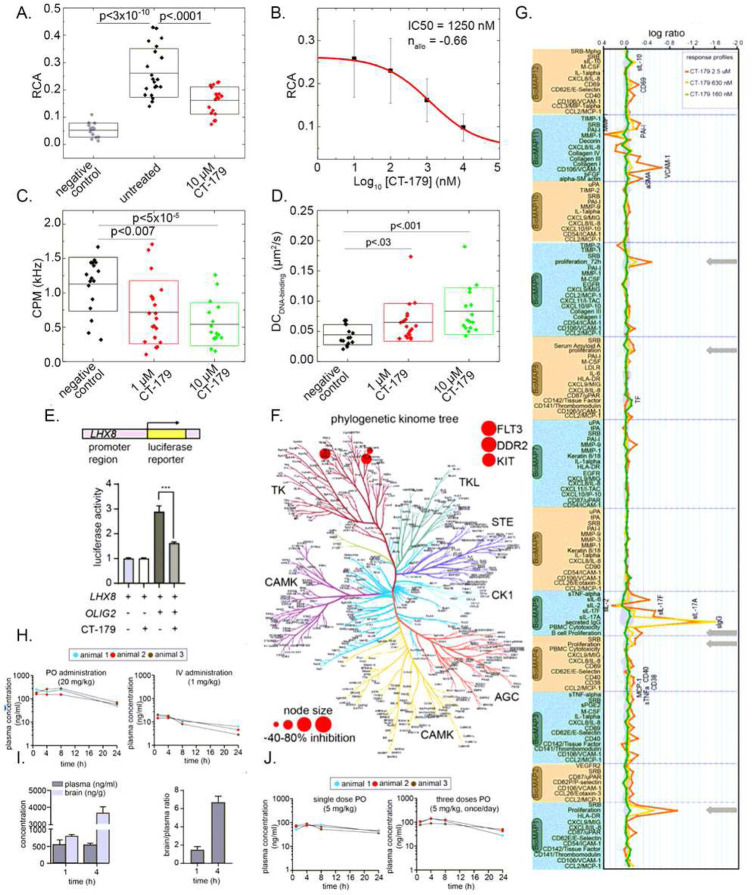
CT-179 specificity analysis and pharmacokinetics analysis. (A) RCA values for replicate HEK-293 cells studied by FCCS. Cells in the negative control (NC) group were co-transfected with *eGFP* and *Tomato* alone. Cells transfected with *OLIG2-eGFP* and *OLIG2-Tomato* showed increased RCA, and treatment with 1 μM CT-179 for one hour significantly decreased RCA in these cells. (B) Increased concentrations of CT-179 resulted in decreased RCA concentrations, yielding a dose-response curve when RCA values measured as in (A) are plotted against CT-179 concentration. Best fit of dose-response curve (red solid line) determined the half maximal inhibitory concentration of CT-179, IC_50_ = 1250 nM, and the allosteric factor, n_allo_ = −0.66. (C) Brightness of OLIG2-eGFP, measured by counts per second per molecule (CPM) in untreated cells and in cells treated with 1 μM or 10 μM CT-179. (D) Diffusion coefficient (DC) of the DNA-binding component. Higher diffusion coefficient, reflecting faster diffusion, was seen in cells treated with CT-179. (E) Luciferase reporter plasmid scheme (top panel) and bar graph showing *LHX8* promoter-based luciferase reporter activity under conditions of OLIG2 overexpression with or without CT-179 treatment. The p value was determined by one-way ANOVA. (F) Kinomic inhibition profile of CT-179. The protein kinases inhibited by CT-179 on the KINOMEscan array are marked as red nodes on the dendrogram of the human kinome. Node size indicates the levels inhibition of the kinases. (G) Phenotypic profiling of CT-179 with BioMAP Diversity PLUS panel. The profile plot shows the effects of CT-179 at 160 nM (orange), 630 nM (yellow) and 2.5 μM (green) on a panel of 12 primary cell systems (listed as BioMAP 1–12) with 148 clinically relevant biomarker readouts. (H) Plasma concentrations of CT-179 measured in 3 animals administered with single dose CT-179 by oral gavage (PO, left) and intravenously (IV, right) over 24 hours. (I) Bar graphs showing plasma and brain concentrations of CT-179 (left panel) and brain to plasma ratio (right) after 1 and 4 hours of single oral administration (PO) of CT-179 at 20 mg/kg. (J) Plasma concentrations of CT-179 over 24 hours after oral administration of drug. The concentration over 24 hours after a single dose (1 dose/day) is shown on the left and after three doses (1 dose/day).

To investigate the therapeutic potential of pharmacologically disrupting OLIG2, we employed CT-179, a small molecule based on the previously described OLIG2 inhibitor SKOG102 [21, 22]. FCCS uses confocal microscopy to quantify interactions between fluorescently tagged molecules in a minute observation volume, in which correlation between different fluorescent signals varies directly with their physical interaction (Supplementary Figure 2A). Prior FCCS studies in live cells showed that SKOG102 disrupts OLIG2 dimerization and DNA binding. We therefore used FCCS similarly to analyze the effect of CT-179 on OLIG2 dimerization using live HEK293 cells co-transfected with OLIG2-eGFP and OLIG2-Tomato fusion constructs. Confocal laser scanning microscopy (CLSM) showed that both fluorescently tagged OLIG2 proteins were localized in the cell nucleus in untreated cells, and that treatment with CT-179 did not change OLIG2 intracellular localization (Supplementary Figure 2B). Corresponding auto- and cross-correlation curves are shown in Supplementary Figure 2C. Larger amplitude of the cross-correlation curve indicated increased interactions between eGFP- and Tomato-tagged OLIG2 molecules relative to eGFP and Tomato tags alone (Supplementary Figure 2B-D). To characterize the strength of these interactions quantitatively, we calculated the relative cross-correlation amplitude (RCA), which reflects on the fraction of complexed OLIG2-eGFP and OLIG2-Tomato molecules. OLIG2-eGFP and OLIG2-Tomato showed significantly higher RCA compared with negative control fluorescent proteins without OLIG2 fusion, when coexpressed in live HEK-293 cells (Figure 2A). The increased RCA of OLIG2-fluorescent protein fusions compared to negative controls demonstrates the ability of the assay to detect OLIG2 dimerization.

Treatment with CT-179 significantly reduced the RCA (Figure 2A), indicating that CT-179 decreased OLIG2 dimerization. The disruption of OLIG2 dimerization increased with CT-179 concentration (Figure 2B), supporting a dose-dependent, causal relationship. As an alternative measure of OLIG2 dimerization and effect of CT-179, we also analyzed OLIG2-eGFP brightness, as reflected by counts per second per molecule (CPM), which reflects OLIG2-eGFP homodimerization. We calculated CPM from the average fluorescence intensity in the green channel divided by the number of OLIG2-eGFP molecules in the observation volume, determined from the amplitude of the autocorrelation curve of the green fluorescence. Brightness in this analysis reflects the number of eGFP molecules complexed together, such that the brightness of an OLIG2-eGFP homodimer is twice that of an OLIG2-eGFP monomer. CT-179 treatment significantly reduced molecule brightness in a dose-dependent manner (Figure 2C), consistent with disruption of OLIG2 dimerization assessed by FCCS.

In addition to these analyses, FCCS provide information about diffusion of OLIG2-eGFP in and out of the observation volume, based on the decay time of the autocorrelation curves. Fitting the data using a 2-component pure diffusion model, two diffusion times could be identified, designated as free OLIG2 diffusion (t_D1_), and OLIG2 diffusion slowed down by its binding to the DNA (t_D2_), which was markedly longer (t_D2_ >> t_D1_). Average diffusion time of free OLIG2-eGFP was determined to be t_D1_ = 700 μs, which was close to the value of free OLIG2-eGFP diffusion in the cell nucleus that was previously determined [22], and was similar to values determined in CT-179-treated cells. In contrast, the diffusion coefficient (DC) of the DNA-binding component increased under treatment with CT-179 (Figure 2D), indicating a shorter residence time of OLIG2-eGFP on nuclear DNA. Similarly, the fractional percentage of the DNA-binding component decreased with CT-179 treatment (Supplementary Figure 2E). These results provide evidence that CT-179 reduced OLIG2-DNA binding, as expected to result from a reduction of OLIG2 dimerization.

To determine if CT-179 interfered with OLIG2 transcriptional activation, we developed a luciferase reporter assay, using Daoy cells to provide a relevant cellular context. Prior ChIP-Seq studies showed that OLIG2 directly interacts with the promoter for *LHX8*, a gene that regulates neuronal differentiation [27]. We transfected Daoy cells with a luciferase reporter fused to the promoter region of the human *LHX8* gene on then measured luciferase expression. Co-transfection with an OLIG2 expression construct induced strong luciferase expression, validating the LHX8 promoter as a reporter of OLIG2-dependent transcription (Figure 2E). CT-179 treatment significantly decreased luciferase expression, demonstrating that CT-179 specifically blocked OLIG2-driven transcription.

### CT-179 shows minimal off-target effects

To detect un-intended inhibitory effects on diverse processes, we used kinome profiling. Testing the effect of 1 μM CT-179 on more than 400 kinases identified 3 kinases that showed potentially significant inhibition, Fetal Liver Tyrosine Kinase 3 (FLT3), Discoidin Domain Receptor Tyrosine Kinase 2 (DDR2) and KIT. DDR2 showed 80% inhibition, FLT3 showed 84% inhibition and KIT showed 66% inhibition (Figure 2F, Supplementary Data 1, Kinome Scan). These kinases were selected for a dose response follow-up assay. The most potently inhibited kinase was FLT3 with an IC_50_ of 20 nM under these assay conditions. However, accounting for intracellular substrate availability using the Cheng-Prusoff equation and assuming 1 mM ATP, the cell potency of CT-179 to inhibit FLT3 is predicted to be IC_50_ > 3.4 μM [28]. We considered this potential inhibition to be negligible because free drug concentrations *in vivo* would be approximately 10 – 100 times lower than the predicted IC_50_. These data indicate that CT-179 does not show any kinase inhibitory effect that would be relevant *in vivo*.

We analyzed the potential for CT-179 off-target toxicity using the BioMAP^®^ Diversity PLUS^®^ platform, a cell-based screen used to assess the safety of pre-clinical experimental therapies on normal healthy tissues. A panel of 12 normal human primary cell lines were included (Supplementary Data 1, BioMAP Readouts). Cells were cultured alone or co-cultured and stimulated with a combination of factors to recapitulate the multi-component signaling networks associated with disease states. CT-179 was profiled at three concentrations (orange, 2.5 μM; yellow, 630 nM; green, 160 nM). The comprehensive panel included 148 clinically relevant biomarker readouts using the 12 primary cell line panel. CT-179 demonstrated 59 annotated effects at 2.5 μM, 24 effects at 630 nM and 11 effects at 160 nM (Supplementary Data 1, PK). At 2.5 μM, which is markedly higher than likely to be achieved *in vivo*, CT-179 decreased proliferation in endothelial cells, T cells, B cells, coronary artery smooth muscle cells and fibroblasts (Figure 2G, grey arrows). At the more biologically relevant concentrations 630 nM and 160 nM, CT-179 displayed minimal off-target effects. Importantly no cytotoxicity was observed even at 2.5 μM. Thus, in kinomic and cytotoxicity assays, CT-179 showed minimal off-target effects.

### In vivo pharmacokinetic studies of CT-179

Two independent CT-179 pharmacokinetic (PK) animal studies, including brain PK, were performed by Biodura Inc. The initial study assessed the bioavailability and concentration versus time of CT-179 in mice administered either a single oral dose (per os, PO) (20 mg/kg) or a single intravenous (IV) dose (1 mg/kg) (Figure 2H). Three mice (C57Bl/J6) were tested in each of the IV and PO groups. Results highlighted consistent plasma concentrations, which were maintained across a 24-hour period, indicating a suitable half-life for once per day dosing. Increasing CNS exposure was detected 4 hours after the initial PO dose, with an estimated brain to plasma (B/P) ratio reaching an average of 6.72 (Figure 2I). The objective of the second study was to estimate steady state exposures of CT-179 in mice receiving PO doses of 1 and 5 mg/Kg (Figure 2J). Results show both PO doses achieved significant plasma levels. CT-179 exposures were measured in the plasma and brain at 24 hours on Days 1 and 3 to compare the initial dose to steady state levels (three doses) (Supplementary Data 1, PK). Findings indicate that CT-179 levels increased approximately 35% between the two time points at the 5 mg/kg PO dose (Supplementary Data 1, PK). CT-179 displayed an estimated brain to plasma ratio of >10, demonstrating CNS penetration and accumulation. While the B/P ratio was well above one, once a steady-state plasma concentration was achieved following repeat dosing, an equilibrium was achieved and the brain tissue concentration did not continue to rise with prolonged exposure. When CT-179 was discontinued, the drug concentration in the brain decreased over time (Supplementary Data 1, PK, 48 hours after Day 3 timepoint). CT-179 displayed a predicted half-life in the range of 10–12 hours and high bioavailability (range estimated as 58–91%; mean 75%) ((Supplementary Data 1, PK). Taken together, the PK studies demonstrated that CT-179 had high oral bioavailability and achieved measurable plasma and brain exposures over 24 hours with somewhat increased exposure between Days 1 and 3 following once daily dosing.

### CT-179 induces apoptosis and mitotic arrest

**Figure 3.**
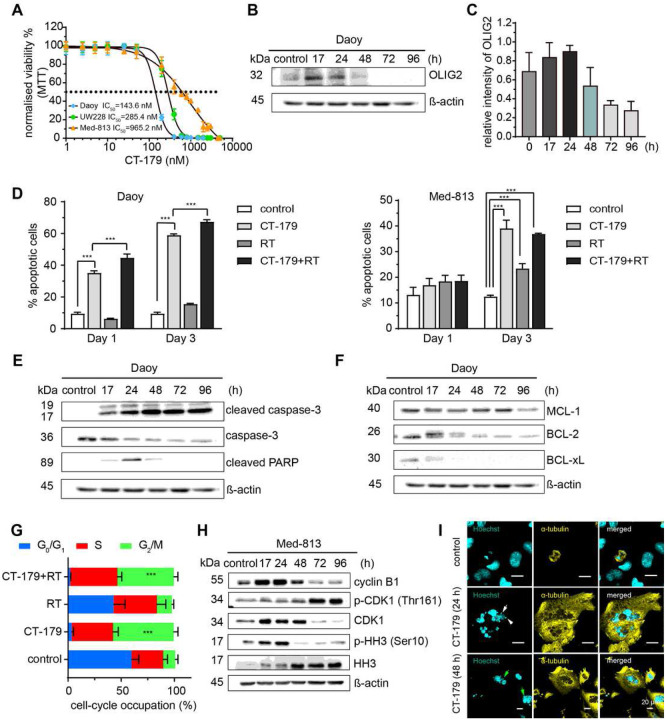
CT-179 induces apoptosis and mitotic slippage in MB cells. (A) MB cell lines were treated with CT-179 for 7 days and a median inhibitory concentration (IC_50_) determined experimentally for each cell line (data are shown as means ± SD, n=3). (B) Daoy were treated with 1 μM CT-179 for 17, 24, 72 or 96 hours, after which total protein were analyzed. OLIG2 expression peaked at 17 and 24 hours after treatment, then decreased to basal and completely diminished after 72 hours. (C) Quantification of OLIG2 expression in Daoy in response of CT-179 (data are shown as means ± SD, n=2). (D) Percentage of Annexin V^+^ cells in Daoy cells and Med-813 treated with CT-179 (1 μM) or RT (2 Gy) alone, or in combination (means ± SD, ***p < 0.001, n=6). The p value was determined by one-way ANOVA. (E) Representative western blots show expression of cleaved caspase-3, caspase-3 and cleaved PARP in Daoy after treated with CT-179 (1 μM). (F) Representative western blots show expression of MCL-1, BCL-1 and BCL-xL in Daoy after treated with CT-179 (1 μM). (G) Cell cycle occupation analysis of Daoy stained with propodium iodide after 24 hours treatment with vehicle or 1 μM CT-179 or RT (2 Gy) alone, or in combination (means ± SD, ***p < 0.001, n=6). The p value was determined by two-way ANOVA. (H) Representative western blots show expression of cyclin B1, p-CDK1, p-HH3, total CDK1 and total HH3 in Med-813 cells after CT-179 (1 μM) treatment. (I) Top panel shows a Daoy cell in anaphase with normal nucleus morphology and spindle alignment. CT-179 treatment (1 μM) results in abnormal nucleus phenotypes including satellite micronuclei (white arrow) and ancillary nucleus lobe formation (green arrows), suggesting cells undergo a mitotic slippage response.

Considering that our FCCS studies of CT-179 confirmed the predicted mechanism of OLIG2 inhibition and that CT-179 showed low off-target effects and the favorable PK, we analyzed the anti-tumor potential of CT-179 in live cells in culture. We selected Daoy, UW228 and Med-813 MB cell lines for initial analysis and treated each line at serial dilutions of CT-179 for seven days. These lines were selected as they showed variable levels of OLIG2 expression. Daoy and UW228 lines showed higher OLIG2 expression in Western blots, compared to Med-813 (Figure 1C), and Daoy cells uniformly expressed OLIG2, while OLIG2 expression in Med-813 cells was heterogeneous (Supplementary Figure 3A). CT-179 showed activity in all 3 lines (Figure 3A) and sensitivities tracked with OLIG2 expression, lowest IC50 in Daoy cells, and highest IC50 in Med-813 cells. Consistent with this trend, cell lines that did not express OLIG2, including HEK293 cells and U87-MG glioma cells were not suppressed by CT-179 (Supplementary Figure 3B). In DAOY cells, OLIG2 protein decreased by 48 hours post CT-179 treatment, indicating specific targeting of OLIG2 (Figure 3B and 3C). CT-179 (1 μM) increased apoptosis, demonstrated by increased cleaved CASPASE-3 (CC3) and sensitized Daoy RT-induced apoptosis (Figure 3D). Reduced expression of cleaved poly-ADP ribose polymerase (PARP) coincided with increasing cleaved caspase-3 overtime, indicating a robust apoptotic response (Figure 3E). The apoptotic response to CT-179 correlated with reduced abundance of anti-apoptotic proteins MCL-1, BCL-2, and BCL-xL (Figure 3F). We also compared the effects on the cell cycle following CT-179 (1 μM) treatment alone versus in combination with RT (2 Gy). Results show a G_2_/M phase arrest following single therapy, which was more pronounced when combined with RT (Figure 3G).

Molecular analysis showed that CT-179 disrupted key mitotic mechanisms. Mitotic cells typically express cyclin B1 at the onset of mitosis when bound CDK1 mediates spindle assembly and mitotic entry [29], then rapidly degrade cyclin B1 after the spindle assembly checkpoint. Simultaneously, cells typically inactivate CDK1 by dephosphorylation and phosphorylate Histone H3 which resolves following telophase [29]. In Daoy cells, CT-179 treatment decreased cyclin B1, CDK1 and p-CDK1, while maintaining persistently elevated p-HH3 (Supplementary Figure 3C). Med-813 responded to CT-179 with a more complex disruption of mitotic mechanisms, with initial increase in cyclin B1, CDK1 and p-HH3 over 24 hours with decreased p-CDK1, followed by a decrease in cyclin B1 and CDK1 and a marked increase in p-CDK1 at later time points (Figure 3H). To explore the effects of CT-179 on nuclear morphology and mitotic spindle formation, Hoechst (cyan) staining of the nucleus and α-tubulin (yellow) staining of mitotic spindles was conducted. IF staining results illustrated cells treated with vehicle had a normal nuclear morphology and spindle alignment, whereas cells treated with CT-179 took on a multinucleated/tetraploid appearance, with satellite micronuclei (white arrow) and an ancillary nuclear lobe formation (green arrow) (Figure 3I). These studies show that CT-179 treatment delayed growth, induced cell killing and resulted in profound mitotic disruption.

### CT-179 treatment induces cell death in MB explant organoids (MBOs)

MB in patients show cellular heterogeneity, and as CT-179 specifically targets the OLIG2-expressing fraction of cells, it is important to evaluate CT-179 in MB models that preserve tissue heterogeneity. For this purpose, we generated explant organoids directly from tumors surgically resected from patients without cell dissociation. This approach allows tumor stroma, blood vessels and immune infiltrate to remain intact. We adapted a recent adult GBM explant approach [30, 31] to freshly resected MB specimens. To the best of our knowledge, we are the first group to adopt this technique to medulloblastoma, generating MBOs that could be cultured for up to 12 weeks.

**Figure 4.**
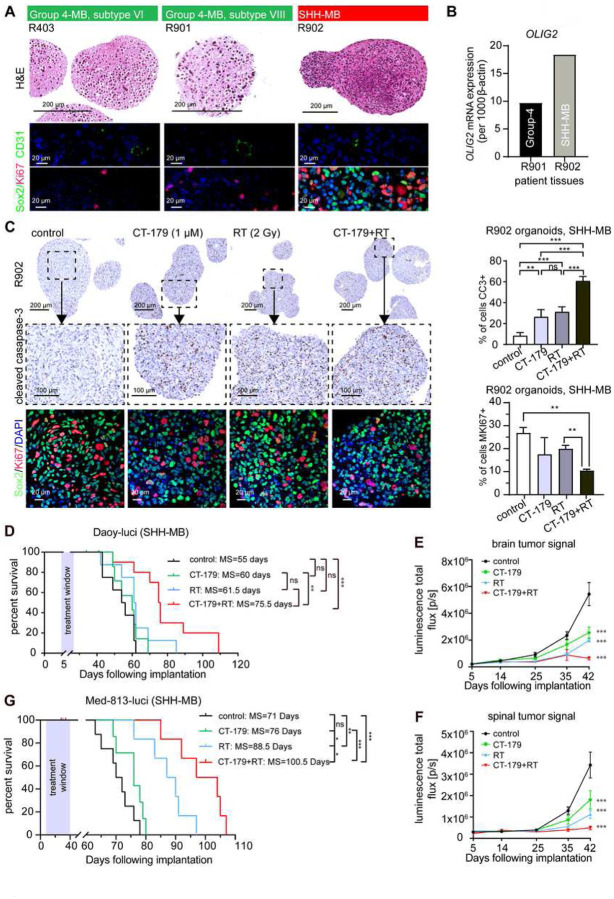
CT-179 induces apoptosis in MB explant organoids. (A) H&E of untreated MB organoids R403, R901 and R902. CD31 staining shows endothelial cells. KI67 staining shows proliferating cells. SOX2, which identified both glial cells and tumor stem cells, is highly expressed in SHH-MB organoid R902. (B) *OLIG2* expression in patient tissues R901 and R902 (means ± SD, n=2). (C) SHH-MB organoid R902 was treated for CT-179 (1 μM) or RT (2 Gy) alone or in combination for 48 hours. IHC shows CC3, KI67 and SOX2 (means ± SD, n=3, *p < 0.05, **p < 0.01, ***p < 0.001). Combination treatment significantly induced cell death in SHH-MB organoids compared to single treatment. The p values were determined by two-sided Student’s t-test. (D) Kaplan-Meier survival curves of Daoy-luci mice (≥ 6 per group, **p < 0.01, ***p < 0.001). MS: median survival. (E) Luminescence signal quantitation of Daoy-luci brain tumors (> 6 per group, ***p < 0.001). (F) Luminescence signal quantitation of Daoy-luci spinal metastases (> 6 per group, ***p < 0.001). (G) Kaplan-Meier survival curves Med-813-luci mice (≥ 6 per group) treated with indicated therapies (*p < 0.05, **p < 0.01, ***p < 0.001) and representative H&E images of Med-813-luci tumor groups. MS: median survival. The p values were determined by Log-Rank test.

DNA methylome profiling classified three selected specimens, R403 (Group 4-MB, subtype VI), R901 (Group 4-MB, subtype VIII) and R902 (SHH-MB). Each of these specimens formed MBOs with heterogeneous morphology, intact blood vessels and proliferative and stem cell populations, demonstrated by IHC for CD31, MKI67 and SOX2 respectively (Figure 4A) [32]. With limitation of the tissue size, we were only able to assess *OLIG2* in two of the tumors, R901 and R902; both had detectable *OLIG2* expression that was greater in R902 (Figure 4B).

We treated R403, R901 and R902 MBOs with CT-179 (1 μM), RT (2 Gy), or both combined for 48 hours. Cleaved caspase-3 staining showed the R403 and R902 MBOs were the most sensitive to CT-179 alone and displayed significant cell death following therapy (Figure 4C, Supplementary Figure 3D). Combination treatment affected the structure of the R403 organoids resulting in gross MBO collapse. The R901 organoid responded similarly to therapy while the MBO structure was more stable (Supplementary Figure 3E). As the response of the SHH subgroup R902 organoid to combination treatment was more pronounced than the Group 4 organoids, we compared the effects of treatment on the fractions of cells in the R902 MBOs that expressed the proliferation marker Ki67 and the glial/stem cell marker SOX2 [33, 34]. These studies demonstrated that combination therapy significantly reduced proliferation without inducing a compensatory increase in the SOX2+ population (Figure 4C).

### CT-179 shows additive anti-tumor effects with radiotherapy in vivo

To determine if CT-179 showed similar efficacy *in vivo* against human MB, we studied CT-179 both as a single agent or combined with RT in immune-compromised NOD *rag* gamma (NRG) mice orthotopically engrafted with Daoy-luci (SHH-MB) and in PDX models Med-813-luci (SHH-MB) and Neo-113 (Group 3-MB). Tumor formation was confirmed by bioluminescence imaging (BLI) (Supplementary Figure 4A,B). We assessed four treatment arms: vehicle control, CT-179 (50 mg/kg, IP twice weekly for two weeks), RT (8 Gy in 2 Gy fractions) and treatment combined. In the Daoy model, one mouse in the CT-179 single treatment arm had skin irritation at the injection site and was censored on Day 28 and another animal showed swelling of the abdomen at the injection site and was censored on Day 35. Median event-free survival (EFS) was 55 days for vehicle control, 60 days for CT-179 alone, 61.5 days for RT alone and 75.5 days for CT179+RT. The increased EFS in the mice in the combination therapy group was statistically significant compared to each other group (Figure 4D). Animals receiving CT-179 + RT treatment progressed noticeably slower, with significantly delayed tumor growth in the brain and spinal cord (Figure 4E,F). Neo-113 engrafted animals were treated with the same regimen as the Daoy model. Log Rank test and Kaplan-Meier survival curves show CT-179 combined with RT significantly prolonged EFS compared to vehicle, while single agent treatment did not result in statistically significant changes (91 days) (**p < 0.01, Supplementary Figure 4C). Given these promising results, we progressed to assessing the primary PDX models Med-813-luci (SHH-MB) using oral gavage, to mimic an oral tablet formulation regime.

We treated Med-813 engrafted animals with 75 mg/kg CT-179 via oral gavage every other day (EOD). With this regimen, CT-179 + RT significantly increased EFS (100.5 days) compared to vehicle (71 days, ***p < 0.001), CT-179 alone (76 days, *p < 0.05) and RT alone (88.5 days, *p < 0.05) (Figure 4G). The prolonged EFS with CT-179 + RT compared to RT alone indicated the ability of CT-179 to increase radiation sensitivity, consistent with the previously reported role of OLIG2+ cells in radiation resistance [17, 18].

We noted toxicities in both the RT group and the CT-179 + RT group (Supplementary Figure 5). Two animals from the RT group and three animals from the combination group were censored due radiation-induced to side effects (Supplementary Figure 4B). These mice displayed weight loss (Supplementary Figure 5A) and enlarged spleens (Supplementary Figure 5B) which were also observed in the toxicity study. Animals presented with slight pallor and hunched posture, histopathological spleen and liver abnormalities (Supplementary Figure 5C,D), decreased red blood cell (RBC) count, hemoglobin (Hgb), and platelets (Plt) were noted in CT-179 + RT group (Supplementary Figure 5E). These toxicities reflect that RT and CT-179 were administered at close to the maximum tolerated dose, and were not incompatible with clinically tolerable brain tumor therapy.

### CT-179 disrupts OLIG2 processing and cell cycle progression in SHH-driven tumors

In an alternative approach to xenograft models that would enable *in vivo* studies of CT-179 in models with typical MB heterogeneity and tumor microenvironment (TME), we analyzed CT-179 treatment in transgenic *Gfap-Cre/SmoM2 (G-Smo)* mice. SHH-pathway hyperactivation in *G-Smo* mice induces MB to form by P10, resulting in tumors with intact immune function and blood brain barrier (BBB), and native TME [18, 35, 36]. MBs in *G-Smo* mice recapitulate the heterogeneity and TME of SHH MB that form in patients [37]. We generated *G-Smo* pups by crossing *GFAP-Cre* and *SmoM2* mouse lines. These animals developed SHH-MB tumors with 100% frequency by P10 as expected [35].

We administered CT-179 via intraperitoneal (IP) injection, testing a range of doses from, 50–150 mg/kg. We found that the maximum tolerated dose (MTD) was 80 mg/kg EOD or 100 mg/kg every three days. Doses above this MTD were associated with brain hemorrhage and early animal deaths (Supplementary Figure 6A,B). In contrast, no hemorrhage was observed at 80 mg/kg EOD or 100 mg/kg every three days.

**Figure 5.**
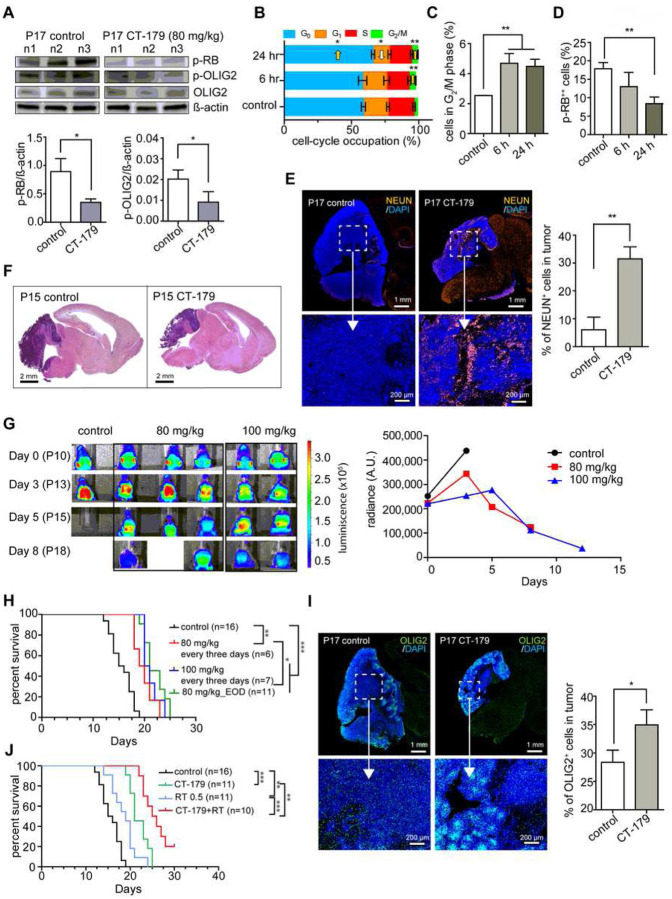
CT-179 efficacy in the *G-Smo* model. (A) Western blots showing p-RB and p-OLIG2 in MBs from replicate *G-Smo* mice treated EOD from P10-P16 with CT-179 (80 mg/kg) or vehicle and harvested at P17, 24 hours after final dose with quantification below. The level of expression was normalized by β-actin (means ± SD, n=3, *p < 0.05). (B) Cell-cycle occupation analysis of MBs from replicate *G-Smo* mice treated with 80 mg/kg CT-179 and harvested after the indicated intervals, dissociated and subjected to flow cytometry, compared to untreated controls (means ± SD, n=3 for each group). (C and D) Quantification by flow cytometry of (C) G_2_/M phase and (D) M phase cells, defined by very high p-RB^++^ cells in dissociated MBs from CT-179-treated mice (means ± SD, n=3, **p < 0.01). (E) Representative NEUN/DAPI IHC in *G-Smo* MB treated with CT-179 100 mg/kg or saline every three days, 24 hours after the final treatment with quantification of NEUN^+^ cells in tumors of replicate mice (means ± SD, n=3, **p < 0.01). (F) H&E of brains P15 *G-Smo* mice treated at P10, P12 and P14 with 80mg/kg CT-179, compared to saline controls. (G) Longitudinal follow up luciferase signal in *G-Smo*^*Gli-luc*^ mice treated as indicated, with luciferase intensity quantified in the right panel. (H) Kaplan-Meier curves of *G-Smo* mice on the CT-179 single-agent regimens (*p < 0.05, **p < 0.01, ***p < 0.001), compared to untreated controls. The p values were determined by Log-Rank test. (I) Representative OLIG2/DAPI IF in MBs from *G-Smo* mice treated EOD from P10-P16 with CT-179 (80 mg/kg) or vehicle and harvested at P17, 24 hours after final dose, with quantification of OLIG2 IF in replicate mice (means ± SD, n=3, *p < 0.05). (J) Kaplan-Meier curves of *G-Smo* mice on the indicated treatment regimens (**p < 0.01, ***p < 0.001), showing the impact of combining radiotherapy and CT-179. The p values were determined by Log-Rank test. For panels A-E and I, the p values were determined by two-sided Student’s t-test.

As an initial test of effect, we administered CT-179 (80 mg/kg) at P10, P12, P14 and P16 and then analyzed tumors by western blot 24 hours after the last dose, comparing phosphorylated OLIG2 (p-OLIG2) as marker of OLIG2 processing and phosphorylated RB (p-RB) as a marker of proliferation. CT-179-treated tumors showed decreased p-OLIG2, consistent with disruption of OLIG2 dimerization, and reduced p-RB, indicating an overall decrease in proliferation (Figure 5A). Reduced p-OLIG2 supports the on-target specificity of CT-179 *in vivo* and reduced p-RB shows that disrupting the OLIG2+ subpopulation produced a detectable anti-tumor effect.

We measured the effect on tumor cell cycle dynamics *in vivo* at 6 and 24 hours after a single 80 mg/kg dose of CT-179. To label cells at S-phase, animals were injected with EdU at 40 mg/kg via IP 30 minutes prior to harvest. We dissociated tumors, stained for EdU, p-RB and DNA content, and quantified each marker by flow cytometry gating as in Supplementary Figure 6C. At 6 hours, CT-179-treated tumors showed significantly increased G_2_/M fractions, consistent with our *in vitro* data. By 24 hours, G_2_/M remained increased, G_0_ also increased and G_1_ decreased, indicating G_0_ and G_2_ arrest (Figure 5B and C). Consistent with G_2_ arrest, mitotic cells, defined by very high p-RB (p-RB^++^) population decreased by 24 hours (Figure 5D). While these studies demonstrate statistically significant growth suppressive effects, the effects were smaller than in our cell line studies, consistent with CT-179 targeting a specific subset of tumor cells.

We analyzed tumor pathology in *G-Smo* mice treated with CT-179 at P10, 12 and 14 and harvested at P15. CT-179-treated tumors showed increased neuronal differentiation compared to controls, demonstrated by increased NEUN expression (Figure 5E). Increased differentiation correlated with reduced tumor growth, as CT179-treated animals consistently displayed smaller tumors at P15 (Figure 5F). These effects *in vivo* show that CT-179 effectively penetrated the blood brain barrier at bioactive concentrations, increased differentiation along regionally appropriate developmental trajectories and slowed tumor growth.

### CT-179 reduces tumor growth in G-Smo mice with SHH-MB

To assess the *in vivo* efficacy of CT-179 longitudinally, we crossed *Gli-luc* mice that carry a GLI-activated luciferase reporter transgene [38] into the *G-Smo* breeders to generate *G-Smo*^*Gli-luc*^ pups. We then compared BLI in *G-Smo*^*Gli-luc*^ mice treated with CT-179 or vehicle administered every three days. Control *G-Smo*^*Gli-luc*^ mice showed an increasing BLI signal over time, reflecting cumulative tumor growth with SHH hyperactivation. In comparison, mice treated with CT-179 showed a dose-dependent decrease in BLI signal (Figure 5G).

To determine if CT-179 produced clinically relevant tumor suppression in the *G-Smo* model, we analyzed the EFS of *G-Smo* mice until symptomatic tumor progression, comparing CT-179 treatment to vehicle-treated controls. Three regimens were tested, CT-179 IP: 80 mg/kg EOD, 80 mg/kg every three days, or 100 mg/kg every three days (Supplementary Data 1, Regimens). Treatment commenced at P10 when tumors were detectable. Treatment continued until progression. All three CT-179 regimens significantly improved EFS compared to control (Figure 5H). Analysis of tumors at P17 showed that the fraction of OLIG2^+^ cells increased in mice treated with CT-179 from P10-P17 (Figure 5I). In the context of reduced p-OLIG2, the increase in total OLIG2 expression suggests a homeostatic response to OLIG2 inhibition that may contribute to the observed resistance in single-agent treatment.

Considering that the bulk of proliferative tumor cells in *G-Smo* MBs were not OLIG2-expressing tumor stem cells, we anticipated that CT-179 would be more effective when combined with a more broadly directed therapy. We therefore assessed the efficacy of CT-179 when combined with RT. Importantly, *G-Smo* mice are radiation resistant and OLIG2+ stem cells contribute to recurrence in these tumors [17, 18]. We treated *G-Smo* mice with RT during CT-179 therapy and compared to RT alone, CT-179 alone, and vehicle-treated controls (Figure 5J). We noted potential for combinatorial toxicity, as initial studies combining CT-179 80 mg/kg EOD with 5 fractions of 2 Gy RT resulted in shortened survival times, and we accordingly adjusted the radiation dose downward (Supplementary Data 1, Regimens). We found that CT-179 plus 3 fractions of 0.5 Gy were tolerable and produced a statistically significant increase in EFS compared to either treatment alone, or to untreated controls (Figure 5J). The addition of RT thus increased CT-179 efficacy, and conversely CT-179 sensitized tumors to RT in a model of highly aggressive and treatment-refractory SHH-MB.

### CT-179 treatment alters MB cellular heterogeneity

As CT-179 specifically targets the OLIG2+ subset of tumor cells in MB, we used scRNA-seq to identify changes in cellular heterogeneity during CT-179 treatment. We treated 4 replicate *G-Smo* mice with CT-179 at P10, 12, and 14 and then harvested tumors on P15. We compared to tumors from 4 replicate, untreated *G-Smo* mice. All tumors were dissociated and processed for split-seq-based scRNA-seq using the Evercode Whole Transcriptome (WT) kit v1 (Parse Biotechnologies). Control replicates showed lower depth of sequencing than CT-179-treated replicates and to facilitate comparisons we randomly down-sampled the data from CT-179-treated replicates by 50% to match the sequencing depth of the controls, in keeping with best practices [39]. 81,208 cells passed filtering and were analyzed, including 54,205 cells from CT-179-treated mice and 27,003 cells from controls.

**Figure 6.**
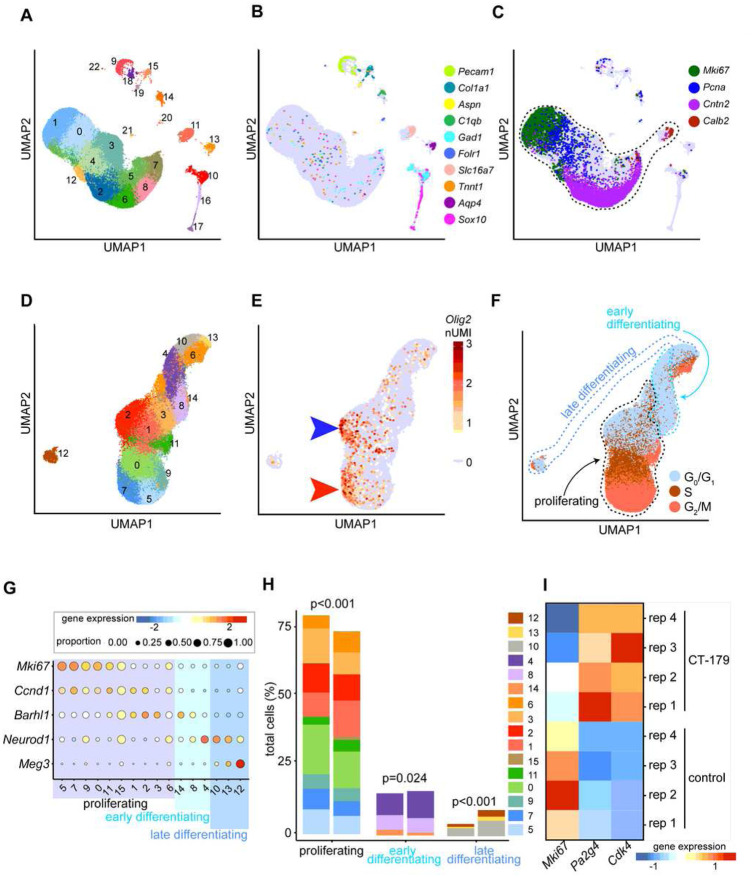
scRNA-seq analysis defines CT-179-induced changes in tumor cell heterogeneity and gene expression. (A) UMAP plot of all cells from CT-179-treated tumors and control tumors, grouped by transcriptomic similarities into color-coded clusters. The clusters are ordered from the largest cluster first (Cluster 0) to the smallest cluster last (Cluster 22). (B) UMAP plot from (A), with expression of specific markers of different types of stromal cells color coded. (C) UMAP plot from (A) with expression of color-coded proliferation and differentiation markers, identifying MB cells in a range of proliferative and differentiating states. The dotted boundary defines the subset of cells of tumor lineage used for further analysis. (D) UMAP from new PCA analysis after isolation of MB cells depicted in (C), showing clustering using PCs from tumor-only analysis. (E) UMAP plot from (D), with expression *Olig2* color coded. *Olig2*+ cells predominantly localize within Cluster 2 (blue arrowhead) and Cluster 7 (red arrowhead). (F) UMAP plot from (D) overlayed with cell cycle phase determined by transcriptomic anlaysis, showing, early and late differentiating cell populations. (G) Dot plot showing the magnitude and frequency of the expression of indicated proliferation and differentiation markers in the indicated tumor cell clusters. (H) Bar plots showing the proportions of proliferative, early differentiating and late differentiating sets of tumor cells color-coded by cluster, comparing CT-179-treated and control tumors. The p values were determined by Dirichelet regression. (I) Heat map showing the expression of *Mki67, Pa2g4,* and *Cdk4* in MBs from individual replicate CT-179-treated or control *G-Smo* mice.

PCA analysis and Louvain clustering based on the first 20 principle components identified 23 clusters (numbered 0–22 from most to least populous). Projected in a 2-dimensional UMAP, discrete clusters formed a large, multi-cluster group, while the other clusters localized more discretely (Figure 6A). We generated cluster-specific sets of differential expressed genes (DEGs) by comparing the expression of each detected by cells within each cluster versus by all cells outside the cluster (Supplementary Data 2). The scRNA-seq data, including expression of individual genes, can be queried at: https://malawskyd.shinyapps.io/ct179_downsampled.

Cell-type specific patterns of gene expression identified clusters comprising various types of stromal cells expected in the brain, including astrocytes, oligodendrocytes, myeloid cells, neurons, endothelial cells, pericytes, fibroblasts, ependymal cells, vascular smooth muscle cells and choroid plexus cells (Figure 6B, Table 1). *Olig2* transcripts were detected in a subset of tumor cells, consistent with the heterogeneous expression shown by IHC [18] (Figure 5I).

To analyze effects of CT-179 on cells of the tumor microenvironment and adjacent normal brain cells, we compared stromal populations in CT-179-treated and control tumors. We normalized the population of each stromal cluster from each replicate to the total number of cells per replicate. To account for the interdependence of the normalized cluster populations within each replicate, used Dirichlet regression to compare CT-179-treated and control groups [40].

CT-179 altered specific stromal populations but did not deplete either of the two oligodendrocyte clusters (Table 1). Mature, myelinating oligodendrocytes showed no significant change while immature oligodendrocytes increased (Table 1). The stability of the population of mature oligodendrocytes and the absence of decrease in immature oligodendrocytes suggest that CT-179 was not toxic to these OLIG2-expressing types of normal cells.

Numerous proliferation markers, exemplified by *Mki67* and *Pcna*, and neural developmental markers, exemplified by *Cntn2* and *Calb2* identified the cells of the large multi-cluster group as MB cells in a range of proliferative and differentiating states (Figure 6C, Table 1, Supplementary Data 2). These states mirrored the developmental trajectory of cerebellar granule neuron progenitors (CGNPs), the cells of origin for SHH-MB differentiating into cerebellar granule neurons (CGNs), as seen in our prior MB scRNA-seq studies [18]. We added to this group the cells of isolated Cluster 11, which expressed CGN marker *Calb2* (Figure 6C) along with numerous neuronal genes (Table 1, Supplementary Data 2), identifying these cells as CGNs, the terminally differentiated pole of the CGNP lineage. To focus on CT-179 effects within the CGNP lineage, we isolated the combined set of MB cells and CGNs for further analysis.

In this more limited set of cells of tumor lineage, we repeated PCA and used this tumor-focused PCA to generate a new set of clusters, cluster-specific sets of differentially expressed genes, and tumor-specific UMAP (Figure 6D, Supplementary Data 3). The related tumor-focused scRNA-seq data, including expression of individual genes, can be queried at: https://malawskyd.shinyapps.io/ct179_downsampled_tumor. *Olig2*-expressing cells comprised a fraction of the tumor population and mapped to two discrete regions, within Cluster 7 and within Cluster 2 (Figure 6E). The detection of *Olig2* mRNA in a relatively small tumor cells was consistent with the heterogeneous pattern of OLIG2 expression in SHH MB PDX tumors (Figure 1D) and previously described in SHH MB GEM models [17].

We projected cell cycle state across the tumor-focused UMAP, using the panel of cell cycle markers provided by the Seurat R Package. (https://cran.r-project.org/web/packages/Seurat/citation.html). This analysis showed that cells at G_0_/G_1_, S and G_2_/M localized to discrete regions (Figure 6F). Comparing the maps of *Olig2* expression and cell cycle showed *Olig2*+ cells divided into quiescent (Figure 6E, blue arrowhead) and actively proliferating subsets (Figure 6E, red arrowhead). This bimodal distribution matched our prior observations of MB stem cells in *SmoM2*-driven tumors [18]. Based on the cell cycle mapping, we identified sets of clusters containing proliferative, early differentiating and late differentiating cells (Figure 6F). Expression of key marker genes confirmed these designations of differentiation state (Figure 6G).

CT-179 treatment altered the distribution of tumor cells across the spectrum of differentiation states. Dirichlet regression analysis showed that CT-179 treatment significantly enriched or depleted different tumor clusters (Table 2), resulting in an overall decrease in proliferative cells and increase in differentiating cells (Figure 6H and 6I). These population shifts were consistent with the changes observed in flow cytometry and IHC studies (Figure 5B and 5E). Together these data show that CT-179 treatment promoted cell cycle exit and completion of neural differentiation.

CT-179 treatment produced complex effects on of G_2_/M phase cells. Our cell cycle analysis identified Clusters 5, 7, 9, 11 and 15 as the full set of clusters enriched for cells in G_2_/M in treated and untreated replicates (Figure 6F). Of these G_2_/M-phase clusters, Clusters 5, 7 and 9 were significantly decreased in CT-179-treated tumors, while both Clusters 11 and 15 increased significantly (Table 2), and Cluster 15 was almost exclusively found in treated tumors (Figure 6I). Cluster 15 showed 5-fold more transcripts per cell compared to the other clusters, indicating a markedly higher cellular mRNA content (p < 1×10^−10^). Based on the expression of G_2_/M phase markers and increased mRNA content, we propose that Cluster 15 represents cells paused at G_2_/M, with consequently more cytoplasm, consistent with the increased G_2_ cells noted in CT-179-treated tumors in our flow cytometry analysis of cell cycle. CT-179 thus altered proliferation dynamics within the tumors, producing complex changes in proliferative sub-populations and a net decrease in proliferative cells. However, proliferative populations persisted after treatment, consistent with the observed development of resistance to single-agent CT-179 therapy and the ultimate progression of all treated tumors.

### Proliferative cells in CT-179-treated MBs up-regulate CDK4

To identify genes that promote proliferation during with CT-179 therapy, we compared gene expression in the proliferative clusters (Figure 6F,G) in CT-179-treated tumors versus controls. We selected the set of genes with corrected p value < 0.5 and expressed in 1.5-fold or greater fractions of cells in CT-179-treated tumors. This analysis identified 106 genes as up-regulated in MB cells that remained proliferative in CT-179-treated mice (Supplementary Data 3). Transcription factor enrichment analysis of using the previously validated ChEA3 Mean Rank tool, [41] identified PA2G4 as the transcription factor with the highest relevance to this set 106 differentially up-regulated genes, with 23 included in both the differential gene set and the set of putative PA2G4 targets determined by ChEA3. Importantly, we did not observe a pattern of gene expression changes consistent with p53-mediated transcriptional regulation, indicating that a DNA damage response was not initiated. Consistent with PA2G4-mediated transcriptional regulation driving the differential gene expression pattern in proliferating cells in CT-179 treated tumors, the *Pa2g4* transcript was within the set differentially up-regulated genes, and was up-regulated in each of the individual CT-179-treated replicate tumors compared to controls (Figure 6I). Moreover, PA2G4 is associated with CDK/RB/E2F signalling [42, 43], and CDK4, another element in the CDK/RB/E2F axis was similarly significantly enriched in cells that remained proliferative in CT-179-treated mice (Table S5). Like *Pa2g4*, *Cdk4* was up-regulated in each CT-179-treated replicate (Figure 6I). Considering the potential for CDK4 to act upstream of PA2G4 via RB/E2F signaling, and the availability of small molecule inhibitors of CDK4 that have been tested in mouse MB models [44–46], we selected CDK4 for further study as a candidate resistance mechanism.

### Palbociclib and CT-179 are mutually enhancing in vivo

We investigated whether CDK4 up-regulation enabled MB growth during CT-179 treatment using a Pox-Palbo, a polyoxazoline nanoparticle formulation of the CDK4/6 inhibitor palbociclib. We previously showed that POx-Palbo suppresses CDK4/6 activity and MB growth more effectively than conventional palbociclib. Similar to CT-179, POx-Palbo produced an anti-tumor effect that was limited by recurrence. We examined whether blocking CDK4 function with POx-Palbo would forestall recurrence during CT-179 treatment.

As an initial step in combining CT-179 with POx-Palbo, we analyzed the effect of POx-Palbo on MB OLIG2 expression. For this purpose, we re-analyzed prior scRNA-seq data from tumors progressing on POx-Palbo therapy (GEO accession# GSE188672). We found that the subset of MB cells previously identified as enriched after chronic POx-Palbo treatment (Supplementary Figure 7A) showed specific expression of stem cell-associated genes (Supplementary Figure 7B,C), suggesting these enriched cells were MB stem cells. We therefore quantified OLIG2-expressing stem cells in tumors recurring during POx-Palbo treatment.

**Figure 7.**
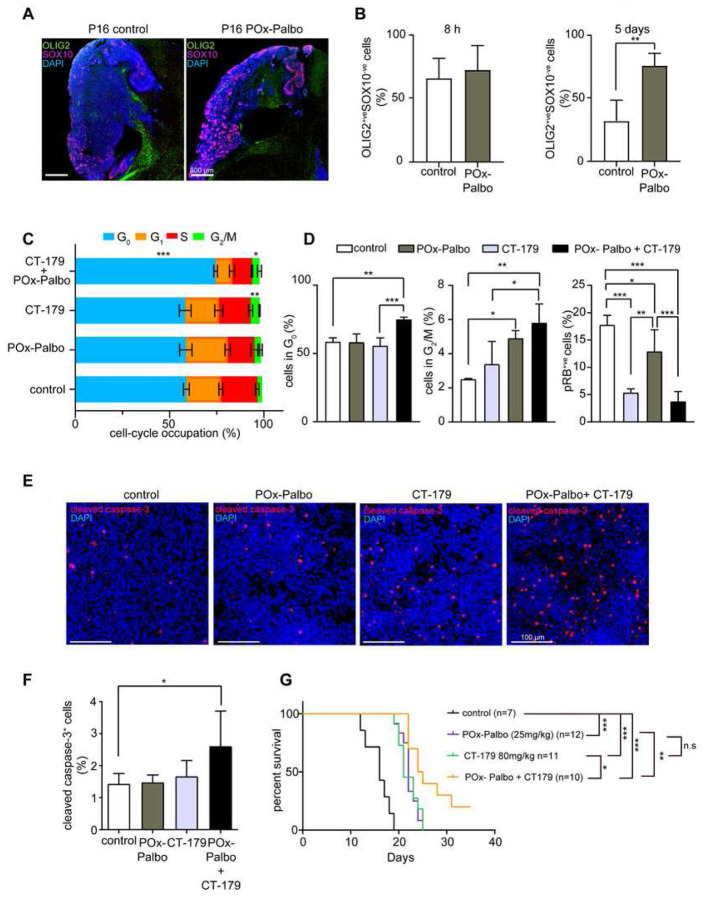
CT-179 and POx-Palbociclib dual regimen is more effective than either single-agent treatment. (A) Representative images of OLIG2/SOX10/DAPI stained MBs in *G-Smo* mice treated with CT-179 (100 mg/kg) or saline every three days. (B) Quantification of OLIG2^+^/SOX10- cells in MBs of *G-Smo* mice after 8 hours (left panel) and 5 days (right panel) of commencing treatment (means ± SD, n>3, **p < 0.01). (C) Cell-cycle occupation analysis of MBs from *G-Smo* mice treated with 80 mg/kg CT-179, 25 mg/kg POx-Palbociclib, or CT-179-POx-Palbo combination and harvested 6 hours after treatment (means ± SD, n=3 for each group). (D) Quantification of percentage of cells in G_0_ phase (left panel) G_2_/M phase (middle panel) and M phase defined by very high phosphorylated (p-RB^++^; right panel) in *G-Smo* mice after 6 hours of treatment (means ± SD, n=3, **p < 0.01). (E) Representative cleaved caspase-3 (cC3)/DAPI stained images from control, CT-179 (80 mg/kg), POx-Palbociclib (25 mg/kg), or combination (POx-Palbociclib + CT-179) treated tumors (F) Quantification of cC3 positive cells. Samples were harvested 6 hours after treatment. (G) Kaplan-Meier curves of *G-Smo* mice on the indicated treatment regimens (*p < 0.05, **p < 0.01, ***p < 0.001). The p values were determined by Log-Rank test. For panels B--D and F, the p values were determined by two-sided Student’s t-test.

As OLIG2 is expressed in MBs by both stem cells and SOX10^+^ oligodendrocytes, we quantified OLIG2^+^/SOX10^−^ cells in palbociclib-treated and control tumors. After 5 days on POx-Palbo therapy, MBs in *G-Smo* mice on sustained CDK4/6 inhibitor therapy showed significantly increased OLIG2^+^ fractions (Figure 7A and 7B), indicating a prominent role for OLIG2-expressing stem cells in driving the growth of recurrent tumors, as seen in recurrence after RT. Tumors thus responded to CDK4/6 inhibition by increasing OLIG2^+^ cells, complementary to the up-regulation of CDK4 in response to OLIG2 inhibition.

To test the potential for simultaneous inhibition of CDK4/6 and OLIG2 to produce a greater anti-tumor effect compared to either intervention alone, we treated replicate *G-Smo* mice with POx-Palbo plus CT-179 and then compared PK effects and efficacy versus CT-179 alone or POx-Palbo alone. For PK studies, we generated replicate P15 *G-Smo* mice treated with POx-Palbo, CT-179, or both drugs together, or no drug, then harvested tumors 6 hours later. The tumors were then divided along the sagittal midline with one half processed for cC3 IHC to quantify apoptosis, and the other half dissociated and subjected to flow cytometry to compare cell cycle distribution and RB phosphorylation between groups. Tumors from mice treated with CT-179 plus PO-Palbo showed increased fractions of cells at G_0_ compared to all other groups (Figure 7C and 7D). Tumors from mice treated with CT-179 alone or CT-179 plus POx-Palbo showed increased fractions at G_2_/M (Figure 7D). However, the fraction of cells with very high p-RB content indicative of M phase was significantly lower in the CT-179 plus POx-Palbo group compared to the CT-179 alone group (Figure 7D), and together increased G_2_/M with decreased high p-RB indicates increased fractions of G_2_/M cells paused at G_2_. The tumors treated with CT-179 plus POx-Palbo also showed increased apoptosis (Figure 7E and 7F). The major PK effects of CT-179 treatment, including increased cell cycle exit, increased G_2_ arrest and increased apoptosis were thus all potentiated by blocking CDK4/6 through combination with POx-Palbo.

To compare efficacy of CT-179 plus POx-Palbo to each drug alone, we compared the EFS of *G-Smo* mice treated with both drugs at P10, 11 and 12, and then every other day, to the EFS of mice in our prior studies of CT-179 alone and POx-Palbo alone. Mice on combination therapy showed improved EFS compared to either single agent (Figure 7G). Both the PK studies and the efficacy studies indicate superiority of CT-179 plus POx-Palbo, supporting a model in which OLIG2^+^ cells contribute to MB POx-Palbo resistance and CDK4+ cells contribute to MB CT-179 resistance, and demonstrating the therapeutic potential of targeting OLIG2 and CDK4 simultaneously.

## DISCUSSION

Paediatric brain cancer accounts for significant morbidity and mortality among childhood cancer sufferers. MB requires aggressive radiotherapy and chemotherapy to minimize recurrence, but at the cost of long-term cognitive, psychosocial and medical complications. Novel therapies are needed to reduce recurrence and lessen disease burden. In this study, we demonstrate that the OLIG2 inhibitor CT-179 can target OLIG2-expressing tumor stem cells that are resistant to conventional therapies, and thus increase the efficacy of multi-modal therapy, most prominently in SHH-driven MB. These studies show that CT-179 has the potential to enable new regimens that will be less prone to recurrence.

The strategy of targeting of OLIG2-expressing cells was suggested by many lines of evidence. OLIG2-expressing MB cells divide rapidly during the initial phase of tumorigenesis and then become a quiescent reservoir of stem cells that can drive recurrence after therapy [17, 18]. Studies of OLIG2 protein expression in MB show foci of OLIG2 positive cells in 75% of cases [47, 48]. Both classic MB (40%) and desmoplastic MB (77%) histological subgroups exhibit OLIG2 positivity [48]. Our analysis of *OLIG2* mRNA expression and survival times in MB patients demonstrated a correlation between OLIG2 expression and recurrence risk that was specific to the SHH-subgroup. These data support OLIG2 targeting as a new approach to MB therapy, particularly for SHH-subgroup patients.

Interference with dimerization presents a specific and effective approach to disrupt transcription factor activity. However, since the protein-protein interaction surface in transcription factor dimerization may be large, designing interfering agents requires detailed molecular modelling and validation. Our FCCS studies show that CT-179, like the template molecule SKOG102 [21, 22], effectively disrupted OLIG2 dimerization, validating its potential to act as a specific OLIG2 inhibitor. The on-target effectiveness of CT-179 was further demonstrated in our *in vitro* LHX8 reporter assays and by our studies of OLIG2 phosphorylation *in vivo*. Alongside these on-target effects, CT-179 showed minimal off-target effects in cell toxicity screens and kinomic assays and did not induce a p53 signal in *G-Smo* tumors analyzed by scRNA-seq. Thus, CT-179 showed on target disruption of OLIG2 and minimal off-target effects at the doses tested.

We studied CT-179 efficacy *in vitro* using cell lines and clinically relevant explant MB organoids, and *in vivo* using PDX and GEM models. Treatment of OLIG2-expressing MB cell lines *in vitro* with CT-179 resulted in G_2_ arrest, increased apoptosis and decreased OLIG2 expression. CT-179 induced gradual degradation of cyclin B1 and p-CDK1 and caused cells to exit mitosis without dividing chromosomes into anaphase. Consistent with our IF staining results, tetraploid MB cells were observed after treatment that exited mitosis through mitotic slippage [49]. Protein expression analysis demonstrated accumulation of cleaved PARP 24 hours post treatment onwards, indicating rapid and significant cell death *in vitro*. These studies in cell lines, which were comprised largely or entirely of OLIG2+ cells, show the impact of OLIG2 disruption in OLIG2-expressing cells.

As SHH MB in patients show more heterogeneous OLIG2 expression, we studied MB tumor explant organoids (MBOs), which recapitulate key-aspects of tumor heterogeneity *in vitro*. Recently, Jacob and colleagues reported the generation and bio-banking of patient-derived GBM 3D explant organoids, known as GBOs [30, 31]. Most 2D and 3D *in vitro* model systems do not preserve the tumor microenvironment, blood vessel structure and immune infiltrate due to tissue digestion and cell dissociation. GBOs are generated with minimal perturbation, fully maintaining heterogeneity for up to 12 weeks. The utility and clinical relevance of GBOs is increasingly being recognised [30]. To the best of our knowledge, we are the first group to have successfully generated MBOs using the Jacob et al approach. We generated three MBOs which displayed morphology faithful to the original tumor. Subsequent testing with CT-179 alone or in combination with radiation showed increased percentages of cleaved caspase-3-positive cells and decreased Ki67-positive cells providing evidence of efficacy in a relevant model-based system of patient-derived MB tumor.

Orthotopic xenograft and PDX models enabled *in vivo* testing of CT-179 efficacy in human MB. To mimic an oral regime, CT-179 was administered by gavage in our primary PDX model experiments. In the PDX models, we noted that CT-179 prolonged survival with tolerable toxicity when used in combination with RT. Consistent with a specific role for OLIG2 in SHH-MB, CT-179 produced greater responses in the SHH-driven Daoy and Med-813 than in the Group-3 Neo-113 model. This difference in efficacy was expected based upon the correlation between *OLIG2* and worse prognosis in patients with SHH-MB. However, OLIG2-expressing cells are also found in other subgroups [23] and that OLIG2 may contribute to tumor heterogeneity across MB disease subsets [50], which may explain the response observed in the Group-3 Neo-113 model.

We used the *G-Smo* GEMM model of SHH-MB to study *in vivo* CT-179 efficacy in tumors with heterogenous OLIG2 expression. This model features an intact BBB [36] and cellular heterogeneity that resembles SHH-MB in patients [37], including heterogeneous *Olig2*+ expression [18]. However, unlike most MBs that occur in patients, the *G-Smo* tumors are refractory to conventional therapy [18]. In *G-Smo* mice, CT-179 showed statistically significant cell cycle disruptions, including increased G2 arrest that that resembled the effects seen Daoy cells *in vitro*. However, fewer G-Smo cells were altered, consistent with CT-179 acting specifically on the *Olig2*-expressing subset. studies, CT-179-treated *G-Smo* tumors also showed altered OLIG2 phosphorylation, demonstrating effective targeting of OLIG2 processing. CT-179 reduced proliferation in *G-Smo* tumors and induced terminal differentiation, demonstrated by p-RB and NEUN+ studies, and by scRNA-seq analysis. CT-179 treatment slowed tumor progression, demonstrated by BLI in *G-Smo*^*Gli-luc*^ mice, and prolonged survival of mice with MB. Together our studies in GEMM and PDX models demonstrate significant anti-tumor effect of CT-179.

Consistent with CT-179 targeting a subset of the proliferative tumor cells, all *G-Smo* mice eventually progressed on single-agent CT-179 therapy, indicating the need for CT-179 to be combined with additional therapeutic modalities. The combination of CT-179 plus RT was more effective than either treatment alone, indicating that in the radio-resistant *G-Smo* model, CT-179 was able to enhance RT efficacy and forestall recurrence. The scRNA-seq analysis of tumors treated with CT-179, moreover, identified numerous transcriptomic changes in cells that remained proliferative, including increased expression of PA2G4-regulated genes and CDK4. As CDK4 is a readily druggable target, we were able to show that CDK4 up-regulation contributed functionally to recurrent tumor growth, as blocking CDK4 enhanced CT-179 efficacy. CT-179 thus integrated well into multimodal regimens with mutual potentiating effects.

OLIG2 plays a critical role in oligodendrocyte function [51] which is essential for brain development [52], and the safety of inhibiting OLIG2 is an important consideration. SHH-MB tumors constitute the pre-dominant tumor type in young children (<3 years of age) as well as in adults (>17 years of age) [3, 53]. The <3 years of age cohort, in-particular is at a stage of significant physiological and neurological development, and humans display prolonged myelination well beyond adolescence [54]. Whether CT-179 produces clinically significant myelin toxicity will need to be evaluated. However, OLIG2 function in oligodendrocytes may be fundamentally different from OLIG2 function in tumor cells, where it modulates the chromatin landscape to activate a unique oncogenic program [55]. CT-179 may, therefore, specifically act on tumor cells without harming normal brain. Consistent with this possibility, scRNA-seq analysis showed no change in the size of the population of myelinating oligodendrocytes, and no depletion of immature oligodendrocytes, which were increased by CT-179 treatment. While additional studies of myelination will be needed, our data suggest that CT-179 will not be toxic to myelinating cells.

In summary, this study shows that inhibition of OLIG2-positive MB tumor cells in combination with RT significantly slows MB progression *in vivo*. CT-179, a novel, small molecule brain penetrate OLIG2 inhibitor, holds significant promise, particularly for the treatment of SHH-driven MB. CT-179 was recently awarded FDA Rare Paediatric Disease Designation for the treatment of MB in September 2020, paving the way for clinical testing of this effective OLIG2 inhibitor in children with SHH-driven MB.

## STAR METHODS

### LEAD CONTACT

Further information and requests for resources and reagents should be directed to and will be fulfilled by the Lead Contact, Prof Bryan Day (Bryan.Day@qimrberghofer.edu.au).

### MATERIALS AVAILABILITY

All unique/stable reagents generated in this study are available from the Lead Contact with a completed Materials Transfer Agreement.

#### Data availability

All scRNA-seq data have been deposited in the publicly available GEO database, under accession number GSE233519.

### METHOD DETAILS

#### Animal models

PDX models of patient-derived medulloblastomas in mice were approved by the human ethics committee of the Queensland Institute of Medical Research (QIMR, Brisbane, QLD), Queensland Children’s Hospital (QCH) and Queensland Children’s Tumor Bank (QCTB) as protocols P3420-A2102–601M and P2324-A1706–612M. Female 6–7 week-old NOD-*Rag1*^*null*^ *IL2rg*^*null*^ (abbreviated as NOD *rag* gamma, NRG) mice were used for toxicity and efficacy experiments. To assess the toxicity of CT-179, CT-179 (80 mg/kg) or saline was given to mice via oral gavage followed by RT (total 8 Gy, 2 Gy per fraction). Mice were monitored closely during the treatment. All mice were euthanised 4 days post treatment. Spleens and livers were collected and fixed in 10% neutral buffered formalin solution. Mouse blood was collected by cardiac puncture. The blood was analyzed on a haematocytometer (Beckman Coulter).

To assess the efficacy of CT-179, mice received stereotactic-guided injection of live Daoy-luci cells (1 × 10^5^), Med-813-luci cells (3 × 10^5^) and Neo-113 filter tissue (FT). The stereotactic coordinates used to target the cerebellum, the orthotopic site of MB tumors, is as follows: 0.8 mm posterior to lambda, 1 mm lateral to the sagittal suture and at a depth of 2.5 mm. The Daoy and Med-813 models have been engineered to constitutively express the firefly luciferase gene (luci). Treatment started after tumor formation confirmed by bioluminescent imaging using the Xenogen IVIS^®^ Spectrum system (PerkinElmer, USA). CT-179 (50 mg/kg via IP, 75 mg/kg via oral gavage) or saline was given to mice followed by RT (total 8 Gy, 2 Gy per fraction). Tumor progression was monitored using the Xenogen IVIS^®^ Spectrum system. As per QIMR ethical guidelines, mice were sacrificed upon signs of tumor burden.

Medulloblastoma-bearing mice were maintained at UNC in accordance with UNC IACUC protocols 19–098 and 21–011 and at QIMR under protocol 1572 and 2324. Specimens were examined by a neuropathologist to verify tumor type and grade.

#### Cell culture

MB cell lines Daoy, UW228, and D283 were obtained from the American Type Culture Collection (ATCC) and cultured in RPMI supplemented with 1% Penicillin-Streptomycin, 1% GlutaMax-I CTS (Gibco) and 10% fetal bovine serum (FBS) (Gibco). MB002, D425 and PER547 primary cell lines were kindly provided by Dr. Tobias Schoep (Telethon Kids Institute, WA). Med-2312 PDX, Neo-113 PDX, Med-2112 PDX, Med-113 PDX and Med-813 PDX were filter tissues purchased from the Olson Laboratory (https://research.fhcrc.org/olson/en/btrl.html). Cell lines derived from the corresponding PDX tissues were labelled as primary cell lines. MB-R201 PDX is a MB PDX model generated and established in our laboratory. MB-R203 is a primary MB cell line. Primary cell lines were derived from MB patient specimens and cultured either in serum-free media grown as spheres or on matrigel [56]. The serum-free media was prepared as previously described [57]. Fresh surgically resected MB tissues were obtained from Queensland Children’s Tumor Bank. MB explant organoids (MBOs), R403 and R901, were generated by microdissection under a stereomicroscope (Zeiss) within a laminar flow biosafety cabinet. The MBOs were cultured in glioblastoma organoid (GBO) medium modified based on Jacob et al. [31]. All cell lines were incubated at 37 °C under 5% CO_2_/95% humidified air atmosphere. All cell lines used in this study were authenticated by short-tandem repeat profile and tested for absence of mycoplasma contamination.

#### FCCS- Acquisition of data

##### Human Embryonic Kidney HEK293 cell culture

HEK293 cells were maintained in a humidified atmosphere containing 5 % CO_2_ at 37 °C in Dulbecco’s Modified Eagle Medium supplemented with 10 % fetal bovine serum (FBS) and antibiotics (100 U/mL penicillin and 100 μg/mL streptomycin). One day before the transfection, HEK293 cells were split into Lab-Tek 8-well chambered Coverglass (Thermo Fisher Scientific). HEK293 cells on and 8-well chamber were transfected with plasmid DNA (OLIG2-eGFP and OLIG2-Tomato; or with eGFP and Tomato as negative control) using Lipofectamine 2000 (Thermo Fisher Scientific). After the transfection, HEK293 cells were cultured for 24 h at 37 °C.

CT-179 was dissolved in MQ water and diluted to 1 mM CT-179 stock solution at 4 °C. The CT-179 stock solution was further diluted with phenol red free medium, for treatment of transfected HEK293 cells for 1 h at 37 °C.

##### Confocal Laser Scanning Microscopy (CLSM) Imaging and FCCS measurements

CLSM imaging and FCCS measurements were performed using an LSM880 microscope system (Carl Zeiss, Germany) equipped with an Ar-ion laser with three lines: 458 nm, 488 nm and 514 nm, 543 nm He-Ne laser, and a water immersion objective lens (C-Apochromat, 40×, 1.2 N.A., Corr, Carl Zeiss), and a gallium arsenide phosphide (GaAsP) spectral array detector and photomultiplier tube (PMT) spectral array detector. eGFP and Tomato were excited using 488 nm laser and 543 nm laser, respectively. The pinhole size was adjusted to 36 μm. The emitted fluorescence was split by a diffraction grating and detected using a GaAsP detector (wavelength range 496–534 nm for eGFP) and a PMT detector (wavelength range 580–758 nm for Tomato). FCCS measurements in the cell nucleus were repeated 10 times, each measurement lasting 20 s.

#### FCCS- Analysis of data

Data acquired by FCCS was analyzed using the ZEN software (Carl Zeiss). Briefly, auto- and cross-correlation curves were analyzed by fitting equations for two-component free diffusion with one triplet (eq. 1) to the experimentally derived autocorrelation curves and for two-component free diffusion (eq. 2) to cross-correlation curves:

(1) $$G_{i}(\tau )=1+\left(1+\frac{F_{\text{t},i}\cdot e^{\frac{\tau }{\tau_{\text{t},i}}}}{1-F_{\text{t},i}}\right)\cdot \frac{1}{N_{i}}\left[F_{1,i}\cdot {\left(1+\frac{\tau }{\tau_{\text{D}1,i}}\right)}^{-1}\cdot {\left(1+\frac{\tau }{S_{i}\cdot \tau_{\text{D}1,i}}\right)}^{-\frac{1}{2}}+\left(1-F_{1,i}\right)\cdot {\left(1+\frac{\tau }{\tau_{\text{D}2,i}}\right)}^{-1}\cdot {\left(1+\frac{\tau }{S_{i}\cdot \tau_{\text{D}2,i}}\right)}^{-\frac{1}{2}}\right]$$

(2) $$G_{c}(\tau )=1+\frac{N_{c}}{N_{g}\cdot N_{r}}\cdot [F_{1,c}\cdot {\left(1+\frac{\tau }{\tau_{\text{D}1,c}}\right)}^{-1}\cdot {\left(1+\frac{\tau }{S_{i}\cdot \tau_{\text{D}1,c}}\right)}^{-\frac{1}{2}}+\left(1-F_{1,c}\right)\cdot {\left(1+\frac{\tau }{\tau_{\text{D}2,c}}\right)}^{-1}\cdot {\left(1+\frac{\tau }{S_{c}\cdot \tau_{\text{D}2,c}}\right)}^{-\frac{1}{2}}]$$

where, *i* denotes OLIG2-eGFP or OLIG2-Tomato and *c* cross-correlation, *F*_t_ is fraction of triple state, *t*_t_ is the relaxation time of the triple state, *N* is the number of molecules, *t*_D1_ and *t*_D2_ are diffusion time of the first and second component, respectively, *F*_1_ is the fraction of first component (*F*_1_+*F*_2_ = 1), *S* is structure parameter.

Diffusion time of the first component, which reflects free diffusion of OLIG2 fluorescent protein fusion construct, was first determined from all measurements presented in this study, and then fixed to the determined value, τ_D1_ = 700 μs, to precisely quantify the diffusion time and fraction of the DNA-bound component.

Values of the lateral, ω_*i*_, and axial, z_*i*_, radii were determined in calibration measurements using ATTO488 (D_ATTO488_; 400 μm^2^s^−1^) and Rhodamine B (D_RhB_; 427 μm^2^s^−1^).

(3) $$\omega_{j}=\sqrt{4D_{j}\cdot \tau_{\text{D},j}}$$

(4) $$Z_{j}=S_{j}\cdot \omega_{j}$$

where, *j* denotes ATTO488 or Rhodamine B, *D* is diffusion coefficient, t_D_ is diffusion time, and S is the structure parameter determined in calibration measurements.

Diffusion coefficient of OLIG2-eGFP slowed down by DNA-binding, D_DNA-bound_, was calculated as follows:

(5) $$D_{\text{DNA-bound}}=\frac{\omega_{\text{ATTO488}}^{2}}{4\cdot \tau_{\text{OLIG2-eGFP, DNA-bound}}}$$

To assess the interaction between OLIG2-eGFP and OLIG2-Tomato, relative cross-correlation amplitude (RCA) which is the ratio of the number of bound molecules and the total, free and bound, number of OLIG2-eGFP molecules:

(6) $$RCA=\frac{N_{\text{c}}}{N_{\text{OLIG2-eGFP }}}$$

To support FCCS data, we also calculated from the autocorrelation curves and average count rates OLIG2-eGFP brightness (as reflected by counts per second per molecule (CPM)) which is reporting on OLIG2 homodimerization:

(7) $$CPM_{\text{OLIG2-eGFP}}=\frac{FI_{\text{OLIG2-eGFP}}}{N_{\text{OLIG2-eGFP}}}$$

where, FI denotes average fluorescence intensity, *i.e*. average count rate and N is the average number of OLIG2-eGFP in observation volume determined from the amplitude of the corresponding autocorrelation curve.

#### FCCS- Software and statistical analysis

Statistical analysis was performed using 2-sided Student’s *t*-test in Microsoft Excel. Dose-response curve fitting was performed using OriginPro 2018, Data Analysis and Graphing Software (OriginLab Corporation, USA).

#### Reverse transcription and quantitative Real-Time PCR

Total RNA was extracted from cell lines and tissue samples using RNeasy Mini Kit (Qiagen, USA) according to the manufacturer’s instructions. First-strand cDNA was synthesized using Superscript IV (Invitrogen). Relative quantitation of gene expression was determined by real-time PCR which was carried out using SYBR^®^ Green PCR Master Mix (Applied Biosystems) following the manufacturer’s instructions. All reactions were performed in duplicate on an ABI Viia 7 (Applied Biosystems). Cycle thresholds (Ct) were determined and exported using QuantStudio^™^ software (Applied Biosystems). The mRNA transcripts levels of genes of interests were determined by the relative expression to the β-actin reference gene. Primers used are listed in the Key resources table.

#### Biomap Diversity PLUS assay

This assay was used to evaluate if CT-179 has off-target effects in the Diversity PLUS panel which consists of 12 BioMAP systems containing early passage primary human cell types from multiple tissues that represent a broad range of human biology relevant to multiple therapeutic areas. The experiment was done by Curtana Pharmaceuticals. The CT-179 was evaluated at 0.15, 0.62 and 2.5 μM. Cells were cultured alone or as co-cultures and were stimulated with a combination of factors, including cytokines, growth factors, and mediators, to recapitulate the multi-component signalling networks associated with disease sates.

Diversity PLUS panel is listed in Table S1. The responds of the Diversity PLUS panel to the CT-179 was calculated as

$$\text{log}10\frac{\text{CT}-179}{\text{ Vehicle control}}$$

The Supplemental Table 1 listed the hits table with the number of biomarker activities that are outside of the 95% significance envelope of vehicle controls.

#### KINOMEscan assay

Effect of CT-179 on protein kinase activity was evaluated using the Thermofisher Scientific SelectScreen Kinase Profiling Service. Over 400 human protein kinases were tested in the presence of 1 μM final concentration of CT-179 at an ATP concentration equal to the known ATP Km for each kinase. The experiment was done by Curtana Pharmaceuticals. Data is presented as percentage of inhibition of enzyme activity. Software CORAL was used to make the kinome tree, which is available from: http://phanstiel-lab.med.unc.edu/CORAL/.

#### Luciferase reporter assay

Promoter region of human *LHX8* was amplified by PCR from human genomic DNA extracted from Med-813luci cells with primers shown in STAR METHODS. The promoter region was inserted into the pGL3-Basic luciferase-reporter vector (Promega). Flag-OLIG2 were synthesised as a gBlock flanked by the Nhel and BamHI restriction enzyme sites (Integrated DNA Technologies, Singapore). The gBlock was restriction digested and ligated into the expression vector, pLEX_307 (a gift from Joseph Rosenbluh, Monash University, Australia) to form pLX307/FLAG-OLIG2. Firefly luciferase-reporter construct (300 ng, pGL3-Basic or pGL3-LHX8), pRL Renilla luciferase SV40 Control Reporter Vector (Promega, 50 ng), and pLX307/Flag-OLIG2 (300 ng) or pLX307/empty vector (300 ng) were reverse co-transfected with Daoy cells (1 × 10^5^/well) in 24-well plates using Lipofectamine^™^ 3000 (Invitrogen). CT-179 (250 nM) was added and incubated for 24 hours. The luciferase activity was examined using Dual-Glo^®^ Luciferase Assay System (Promega) according to the manufacturer’s instructions, and the signal was normalised to vector controls.

The sequence of the Flag-OLIG2 is as follows:

aaagctagccaccatggactacaaagacgatgacgataaagggggcggtggaggtGACTCAGACGCTAGTCTTGTTAGTTCTCGGCCATCTTCCCCTGAGCCGGATGATCTCTTCCTCCCTGCTAGATCTAAAGGGTCATCCGGCAGTGCCTTTACTGGCGGAACTGTGTCCAGCAGCACGCCGTCCGACTGTCCACCCGAACTTTCTGCTGAGCTTCGGGGTGCTATGGGTTCCGCCGGTGCACACCCAGGGGATAAACTGGGGGGCTCAGGATTCAAGTCTTCTAGCAGTAGCACGTCTTCATCCACTTCATCAGCCGCCGCCAGCTCAACAAAAAAAGACAAGAAGCAAATGACGGAGCCCGAGCTTCAACAACTTCGGCTGAAGATCAATAGTAGGGAAAGGAAGCGAATGCATGACCTCAATATTGCTATGGACGGACTGAGGGAGGTCATGCCCTATGCTCACGGACCGTCAGTTCGCAAGCTTAGTAAAATTGCAACACTTCTTTTGGCACGGAACTATATCTTGATGCTGACTAATTCTCTTGAGGAAATGAAACGGCTGGTAAGTGAAATTTACGGTGGACACCATGCAGGCTTTCATCCTTCTGCATGTGGCGGTCTCGCTCATTCAGCACCCCTGCCCGCTGCAACCGCTCATCCCGCTGCGGCGGCACACGCCGCACACCATCCCGCCGTGCATCATCCCATTTTGCCGCCCGCGGCCGCAGCTGCCGCTGCTGCCGCAGCCGCAGCTGCCGTGTCTTCTGCTTCTCTGCCTGGTTCCGGTCTCCCATCTGTCGGCTCTATCCGACCGCCCCACGGTCTGTTGAAATCACCCAGCGCCGCGGCTGCTGCACCCCTTGGAGGCGGTGGTGGAGGGTCCGGGGCCTCCGGGGGGTTTCAGCATTGGGGCGGGATGCCGTGCCCTTGTTCAATGTGTCAGGTACCTCCACCCCACCATCATGTTTCCGCCATGGGAGCAGGTTCCCTTCCTAGACTGACAAGTGACGCCAAGTGAGGATCCaaa

#### In vivo pharmacokinetic studies of CT-179 in mice

The *in vivo* pharmacokinetic studies (EXT-240 and EXT-241) were performed under contract by Biodura Inc.

#### OLIG2 siRNA knockdown

OLIG2 siRNA#1, #2, #3, and scrambled control oligo sequences were purchased from Sigma-Aldrich. OLIG2 knockdown was done using Lipofectamine^™^ RNAiMAX transfection reagent with 30 pmol of siRNA. Knockdown efficiency was confirmed by examining OLIG2 expression at mRNA and protein levels.

#### Irradiation of cells

Cells were seeded into 96-well plates at 10,000 cells/well before irradiation in ^137^Cs source gamma rays to achieve 2 Gy (MDS Nordion Gammacell Irradiator).

#### Cell viability assays

Cell proliferation was determined by a CellTiter 96^®^ Non-Radioactive Cell Proliferation Assay (Promega) kit. Absorbance at 490 nm was measured on a Biotek PowerWave (Biotek, USA) plate scanner to obtain raw data. Average absorbance from triplicate wells were plotted against concentration using GraphPad Prism software v.7.0 (GraphPad software). At the indicated time points, cells were stained using FITC-conjugated Annexin V and Annexin V binding buffer (BD Pharmingen^™^) according to the manufacturer’s instructions. Annexin V or propidium iodide (PI)-positive cells were analyzed using a Fortessa 5 Flow Cytometer (Becton Dickinson) and data was analyzed using FlowJo^®^ software (Tree Star).

#### CT-179 drug treatment

The synthesis of CT-179 is described in published patent application WO2016138479A1. To prepare the drug for oral gavage, CT-179 was dissolved in 0.5% (w/v) methylcellulose 4000/0.5% (v/v) Tween 80 in injection water. To prepare the drug for *in vitro* studies and for intraperitoneal (IP) injection, CT-179 was dissolved in injection water and stored at −80 °C.

#### Cell cycle analysis

Cell cycle analysis was performed as descried previously [58]. Cells were fixed with 70% ice-cold ethanol and subsequently stained with PI and analyzed using a Fortessa 5 Flow Cytometer (Becton Dickinson) and data was analyzed using FlowJo^®^ software (Tree Star).

#### Immunofluorescent staining

Cells were fixed with 4% paraformaldehyde (PFA) then quenched in blocking buffer (0.5% BSA), permeabilisation buffer (1 × PBS/0.3% Triton X-100) and then incubated with Hoechst and α-tubulin (Cell signalling), followed by incubation with Alexa Fluor^®^ 488 secondary antibody. Images were captured using a Zeiss 780-NLO confocal microscope (Zeiss) with a 40 × /1.4 Plan-Apochromat oil lens, and processed using Image J software.

Organoids were fixed in 4% paraformaldehyde (PFA)/ PBS, embedded in 1% low melting point agarose gel and, subsequently, embedded in paraffin. Organoid sections (4 μm) were probed with anti-SOX2 (Abcam), anti-Ki67 (Dako), CD3 (Dako), IBA1 (Abcam), and CD31 (Dako) either in single or in combination and subsequently stained with Mach 2 mouse/rabbit HRP or PE rabbit HRP. Sections were captured using a Zeiss 780-NLO confocal microscope (Zeiss) with a 40 × /1.4 Plan-Apochromat oil lens, and processed using Image J software.

For immunofluorescent staining of mouse MB after CT-179 treatment, brains with tumors were fixed, sectioned, stained and imaged using an Aperio Scanscope as previously described [59].

#### Immunoblot analysis

Whole cell protein lysate (60 μg/sample) was used. Samples were run on denaturing sodium dodecylsulfate-polyacrylamide gel electrophoresis gels (12%) before transferring onto Immuno-Blot^™^ polyvinylidene fluoride membranes (Bio-Rad). The reference protein β-actin was used as a loading control.

#### Flow cytometry-based cell cycle analysis in *G-Smo* tumors

Post-natal P10 *G-Smo* mice were injected IP with CT-179 (80 mg/kg), or POx-Palbo (25 mg/kg, every day from P10 to P13), or combination (CT-179 + POx-Palbo, EOD from P14) or saline control solution and tumors were harvested after 6 or 24 hours. EdU (40 mg/kg) was administered IP 30 minutes before harvest. Tumors were dissociated using the Worthington Papain Dissociation System Kit, then fixed for 15 minutes on ice and washed with FACS Wash buffer (2% FBS in PBS). Fixed cells were stained with fluorophore markers for DNA (FX Cycle stain, Thermo Fisher Scientific), EdU (Click-it Edu Kit, Thermo Fisher Scientific), and phospho-RB content (phospho-RB^Ser807/811^, Cell Signalling Technology) as previously described [59]. Stained cells were resuspended in sheath fluid and ran on LSR II flow cytometer for 50000 events at the UNC Flow Cytometry core, using appropriate compensation controls and analyzed using FlowJo^®^ software (Tree Star).

#### scRNA-seq

##### Split-seq based barcoding and library construction

Tumors from replicate mice were harvested at P15. Tumors were then dissociated with papain as in our prior studies [33] [18] and then processed using the Parse Fixation kit (Cell Fixation Kit v1), according to the manufacturer’s instructions, following the manufacturer’s suggested addition of BSA to prevent cell clumping. Cells were then counted and frozen with 10% DMSO until all replicates from all conditions were collected.

Each replicate was then thawed, and cells were counted and then distributed in the 48 wells of a Parse barcoding plate (Evercode WT (100K) v1), 4 wells per replicate. In wells 47 and 48, human and mouse cell lines were spiked in for QC studies, and these wells were later excluded from the CT-179 versus control analysis and anlayzed separately to confirm the absence of human mice hybrid cells and therefore effective single-cell resolution. Plated cells were then subjected to reiterative bar coding, pooling and re-distribution, according to the Parse protocol [60]. The sublibraries generated through the barcoding protocol were analyzed by TapeStation to confirm DNA size distribution and then prepared for sequencing according to the Parse Barcoding Kit protocol. Sublibraries representing half of the barcoded cells were then sequenced on an Illumina Novaseq6000, using an S2 flow cell, with 86×6×114 paired-end cycles.

##### Data analysis

The scRNA-seq data were analyzed using Parse software to identify cells by bar code, and to map transcripts to the mouse transcriptome. Data were then analyzed using our previously reported pipeline [18, 33].

#### Immunohistochemistry

##### Tissues and organoids

Tissue samples were fixed in 10% neutral buffered formalin and embedded in paraffin and, subsequently, stained with haematoxylin and eosin (H&E). IHC on *G-Smo* mice was as previously described [18]. Antigen retrieval was performed using a pH 9.0 Tris-EDTA buffer. Tissue sections (4 μm) were probed with anti-OLIG2 (Millipore) antibody overnight and subsequently stained with Mach Polymer HRP (Biocare Medical). Organoid sections (4 μm) were probed with anti-cleaved caspase-3 (Biocare Medical) in 10% goat serum for 2 hours at room temperature and subsequently stained with Mach 1 Universal Polymer HRP. Signals were developed in 3,3′-Diaminobenzidine (DAB). Sections were counterstained in Haematoxylin, dehydrated and mounted.

##### GEMM MBs

Brains including tumors from *G-Smo* mice were harvested, fixed in 4% paraformaldehyde for 48 hours, and embedded in paraffin at the UNC Centre for Gastrointestinal Biology and Disease Histology core. Sections were deparaffinised, and antigen retrieval was performed using a low-pH citric acid-based buffer. Staining was performed and stained slides were digitally scanned using the Leica Biosystems Aperio ImageScope software (12.3.3) by the UNC Translational Pathology Laboratory, as in prior studies [18]. The primary antibodies used were anti-NeuN (Cell Signalling Technology) anti-OLIG2 (Cell Marque) and anti-SOX10 (Cell Signalling Technology) and anti-cleaved caspase-3 (Biocare).

### QUANTIFICATION AND STATISTICAL ANALYSIS

*In vitro* experiments - all data represent the means ± standard deviation (SD) or standard error of the mean (SEM) as indicated. Where appropriate, two-tailed Student’s *t*-test or ANOVA were used to determine the probability of difference (*p < 0.05, ** p < 0.01, ***p < 0.001). *In vivo* experiments - the log-rank (Mantel-Cox) test was used to determine the survival significance of survival differences between treatment groups (*p < 0.05, ** p < 0.01, ***p < 0.001). Kaplan-Meier survival curves were generated using GraphPad Prism v. 7.0 software (GraphPad software).

## Figures and Tables

**Table:** KEY RESOURCES TABLE

REAGENT or RESOURCE	SOURCE	IDENTIFIER
Antibodies		
Mouse monoclonal anti-β-actin (clone 8H10D10)	Cell Signalling Technology	Cat# 3700
Rabbit monoclonal anti-β-actin (clone 13E5)	Cell Signalling Technology	Cat# 4970
Mouse monoclonal anti-OLIG2 (clone 211F1.1)	Sigma-Aldrich	Cat# MABN50
Rabbit polyclonal anti-OLIG2	Millipore	Cat# ab9610
Rabbit monoclonal anti-OLIG2 (clone EP112)	Cell Marque	Cat# 387R-14
Mouse monoclonal anti-Bcl-2 (clone 124)	Cell Signalling Technology	Cat# 15071
Rabbit monoclonal anti-Bcl-xL (clone 54H6)	Cell Signalling Technology	Cat# 2764
Rabbit monoclonal anti-Mcl-1 (clone D2W9E)	Cell Signalling Technology	Cat# 94296
Rabbit monoclonal anti-cleaved caspase-3 (Asp175) (clone 5A1E)	Cell Signalling Technology	Cat# 9664
Rabbit polyclonal anti-cleaved caspase-3	Biocare Medical	Cat# CP229
Rabbit polyclonal anti-caspase-3	Cell Signalling Technology	Cat# 9662
Rabbit monoclonal anti-cleaved PARP (Asp214) (clone D64E10)	Cell Signalling Technology	Cat# 5625
Mouse monoclonal anti-cdc2 (clone POH1)	Cell Signalling Technology	Cat# 9116
Rabbit polyclonal anti-phospho-cdc2 (clone Thr161)	Cell Signalling Technology	Cat# 9114
Rabbit monoclonal anti-cyclin B1 (clone D5C10)	Cell Signalling Technology	Cat# 12231
Rabbit polyclonal anti-CD3	Dako	Cat# A0452
Mouse monoclonal anti-CD31 (clone JC-70A)	Dako	Cat# M0823
Rabbit monoclonal anti-Iba1 (clone EPR16588)	Abcam	Cat# ab178846
Mouse monoclonal anti-Ki67 (clone MIB-1)	Dako	Cat# M7240
Rabbit monoclonal anti-phospho-Rb (Ser807/811) (clone D20B12)	Cell Signalling Technology	Cat# 8974
Rabbit monoclonal anti-NeuN (clone D4G4O)	Cell Signalling Technology	Cat# 24307
Rabbit monoclonal anti-SOX2 (clone EPR3131)	Abcam	Cat# 92494
Rabbit monoclonal anti-SOX10 (clone E2V9N)	Cell Signalling Technology	Cat# 78330
Rabbit monoclonal anti-a-tubulin (clone 11H10)	Cell Signalling Technology	Cat# 2125
Goat anti-rabbit horseradish peroxidase	Daka Cytomation	Cat# P0448
Rabbit anti-mouse horseradish peroxidase	Daka Cytomation	Cat# P0260
Critical Commercial Assays		
CellTiter-Glo Luminescent Cell	Promega	Cat# G7572
Cell Fixation Kit v1	Parse Biosciences	Cat# SB1001
Evercode WT (100K) v1	Parse Biosciences	Cat# ECW01030
FITC Annexin V Apoptosis Detection Kit I	BD Pharmingen	Cat# 556547
Superscript IV First-Strand Synthesis System	Invitrogen	Cat# 18091050
RNeasy Mini Kit	Qiagen	Cat# 74904
FxCycle Violet Stain	Thermo Fisher Scientific	Cat# F10347
Click-iT Edu Cell proliferation Kit for Imaging	Thermo Fisher Scientific	Cat# C10337
Experimental Models: Cell lines		
Daoy	ATCC	ATCC Cat# HTB-186
UW228	Dr Tobias Schoep (Telethon Kids Institute, WA)	N/A
D283	ATCC	ATCC Cat# HTB-185
REAGENT or RESOURCE		
Experimental Models: Cell lines		
MB002	Dr Tobias Schoep (Telethon Kids Institute, WA)	N/A
D425	Dr Tobias Schoep	N/A
PER547	Dr Tobias Schoep	N/A
HEK-293	ATCC	ATCC Cat# CRL-1573
Experimental Models: Strains/PDX models		
NOD-Rag1^null^ IL2rg^null^	The Jackson Laboratory	JAX: 007799
C57BL/6 mice	The Jackson Laboratory	Stock #000664
hGFAP-Cre mice	The Jackson Laboratory	
SmoM2-eYFPloxP/loxP mice	The Jackson Laboratory	Stock #005130
Gli-luc mice	Generously shared by Dr. Oren Becher, Northwestern University and Dr. Eric Holland, Fred Hutchinson Cancer Research Center	MGI #4820828
Med-2312 PDX	Dr James Olson (FHCRC)	http://research.fredhutch.org/olson/en/btrl.html
Neo-113 PDX	Dr James Olson	Same as above
Med-2112 PDX	Dr James Olson	Same as above
Med-113 PDX	Dr James Olson	Same as above
Med-813 PDX	Dr James Olson	Same as above
Oligonucleotides	SEQUENCE (5’−3’)	
qRT-PCR: OLIG2 Forward (set 1)	GAAACTACCCCACCGACTCA	
qRT-PCR: OLIG2 Reverse (set 1)	ACCAAACTGTTTCCACAGC	
qRT-PCR: OLIG2 Forward (set 2)	GCTGCGTCTCAAGATCAACAGCC	used for overexpression validation
qRT-PCR: OLIG2 Reverse (set 2)	CGATCTTGGAAAGCTTGCGCAC	
qRT-PCR: GAPDH Forward	GAAGGTGAAGGTCGGAGTCAACG	
qRT-PCR: GAPDH Reverse	GCCATGGGTGGAATCATATTGG	
qRT-PCR: ACTB Forward	CACACTGTGCCCATCTACGA	
qRT-PCR: ACTB Reverse	GTGGTGGTGAAGCTGTAGCC	
NheLHX8 Forward	GTAGCTAGCCCTTAAAAAGGCATCGTATG	
BglLHX8 Reverse	GTAAGATCTGCCCACTTCCGCTGAGCAGC	
OLIG2 siRNA knockdown		Oligo name
OLIG2 siRNA KD#1	Sigma-Aldrich	SASI_Hs01_00092187
OLIG2 siRNA KD#2	Sigma-Aldrich	SASI_Hs02_00092188
OLIG2 siRNA KD#3	Sigma-Aldrich	SASI_Hs02_00092190
MISSION^®^ siRNA scrambled control	Sigma-Aldrich	SIC001
Chemicals		
CT-179	Curtana Pharmaceuticals	Patent application WO2016138479 A1
Software		
Graphpad Prism	GraphPad	http://www.graphpad.com/scientific-software/prism/
FlowJo	Tree Star	http://www.flowjo.com
QuantStudio	Applied Biosystems	https://www.thermofisher.com/au/en/home/global/forms/life-science/quantstudio-3-5-software.html
Aperio ImageScope	Aperio	https://www.leicabiosystems.com/digital-pathology/manage/aperio-imagescope/
OriginPro 2018	OriginLab	https://www.originlab.com/
ZEN SP2.3 SP1 (black edition)	Carl Zeiss	https://www.zeiss.com/microscopy/en/products/software/zeiss-zen.html

## SIGNIFICANCE

We investigate a new approach to medulloblastoma therapy by pharmacologically disrupting the transcription factor OLIG2. Tumor stem cells in SHH medulloblastomas that express OLIG2 can survive conventional therapy and drive recurrence. We show that the small molecule CT-179 blocks OLIG2 dimerization and function, slows tumor growth and combines favorably with radiation and targeted therapies to forestall recurrence.
